# Polymer-Free Versus Biodegradable Polymer Drug-Eluting Stents in Coronary Artery Disease: Updated Systematic Review and Meta-Analysis of Clinical, Angiographic, and OCT Outcomes

**DOI:** 10.3390/biomedicines13061470

**Published:** 2025-06-14

**Authors:** Marcello Marchetta, Stefano Sasso, Vincenzo Paragliola, Andrea Giovanni Parato, Diego De Angelis, Giulio Russo, Giovanni Albano, Daniela Benedetto, Federico Moretti, Francesco Valenti, Gianluca Massaro, Gaetano Chiricolo, Manfredi Tesauro, Giuseppe Massimo Sangiorgi

**Affiliations:** 1 Division of Cardiology, University of Rome “Tor Vergata”, 00133 Rome, Italy; stefanosasso0205@gmail.com (S.S.); vincenzo.uni@gmail.com (V.P.); andreaparato231198@gmail.com (A.G.P.); diegodeangelis97@gmail.com (D.D.A.); giuliorusso.md@gmail.com (G.R.); gioalbano4@gmail.com (G.A.); dania.benedetto@gmail.com (D.B.); f.moretti1997@gmail.com (F.M.); valentifrancesco000@gmail.com (F.V.); gianluca88massaro@gmail.com (G.M.); gaetano.chiricolo@ptvonline.it (G.C.); mtesauro@tiscali.it (M.T.); 2 Division of Cardiology, University of Messina, 98168 Messina, Italy

**Keywords:** meta-analysis, drug-eluting stents, coronary artery disease, percutaneous coronary intervention, polymer-free, biodegradable polymer

## Abstract

**Background/Objectives**: Polymer-free drug-eluting stents (PF-DESs) aim to mitigate long-term adverse effects associated with polymer-based platforms. However, clinical comparisons with biodegradable polymer DESs (BP-DESs) remain limited. The objective of this review is to assess the efficacy and safety of PF-DESs versus thin-struts (<100 μm) BP-DESs in patients undergoing percutaneous coronary intervention (PCI). **Methods**: We conducted a systematic review and meta-analysis of randomized controlled trials (RCTs) comparing PF-DESs and BP-DESs in adults undergoing PCI. PubMed, Embase, and CENTRAL were searched up to 1 February 2025. A random-effects model was used to calculate pooled risk ratios (RR) or mean differences (MD) with 95% confidence intervals (CI). Outcomes included myocardial infarction (MI), all-cause and cardiac death, target lesion revascularization (TLR), stent thrombosis, and angiographic/OCT parameters. Subgroup and sensitivity analyses were conducted for outcomes with high heterogeneity (I^2^ > 50%). **Results**: Nine RCTs (*n* = 9597) were included. At 12 months, no significant differences were found between PF-DESs and BP-DESs for TLR (RR 1.51; 95% CI: 0.83–2.75), MI, or stent thrombosis. At 24 months, MI and all-cause death were similar between groups. A subgroup analysis showed lower cardiac death with the BioFreedom stent (RR 0.57; 95% CI: 0.35–0.90), not observed in non-BioFreedom devices. No significant differences were detected in angiographic or OCT outcomes, though heterogeneity was high. **Conclusions**: PF-DESs and BP-DESs demonstrated comparable clinical performance. The observed benefit in cardiac death with BioFreedom may reflect device-specific effects and merits further investigation.

## 1. Introduction

Drug-eluting stents (DESs) represent the current standard of care for percutaneous coronary intervention (PCI) [1]. However, concerns persist regarding long-term adverse outcomes, including delayed healing, chronic inflammation, neoatherosclerosis, and late or very late stent thrombosis, largely attributed to the durable polymers traditionally employed for drug release [2]. To mitigate these risks, newer generation DESs featuring biodegradable polymers (BP-DESs) and polymer-free platforms (PF-DESs) have been developed. Polymer-free stents theoretically offer several distinct advantages, primarily related to the elimination of the polymer coating, which is implicated in persistent vessel inflammation and hypersensitivity reactions [3]. Furthermore, polymer-free designs may enhance endothelialization and potentially reduce thrombogenicity, thus theoretically improving long-term vessel healing and clinical outcomes [4]. Another critical technical aspect influencing stent performance is strut thickness [5]. Previous studies have consistently shown that thinner struts (<100 µm) significantly reduce the risk of in-stent restenosis, compared to thicker struts (>100 µm). In particular, randomized controlled trials (RCTs) have demonstrated substantial reductions in angiographic restenosis and restenosis-related reinterventions associated with thin-struts stents [6,7]. Previous meta-analyses comparing polymer-free DESs with polymer-based DESs have yielded heterogeneous findings, indicating comparable safety and efficacy outcomes overall, but also emphasizing the need for updated evidence given the continuous evolution in DES technologies [8]. Specifically, the most recent meta-analysis directly comparing polymer-free and biodegradable polymer DESs [9] underscored an ongoing uncertainty in clinical outcomes, as several RCTs have been published subsequently, potentially influencing the balance of evidence. To date, no comprehensive synthesis has incorporated the most recent randomized evidence comparing polymer-free DESs with newer-generation biodegradable polymer DESs under strict control for strut thickness—a technical feature that critically modulates clinical performance. Therefore, the aim of this systematic review and meta-analysis was to update the existing evidence base, incorporating the latest randomized trials, to directly compare the clinical efficacy and safety of PF-DESs versus new-generation BP-DESs in patients undergoing PCI for coronary artery disease CAD, providing a qualitative and quantitative synthesis of clinical, angiographic and optical coherence tomography (OCT) outcomes.

## 2. Methods

### 2.1. Study Design and Reporting Guidelines

This systematic review and meta-analysis adhered to the Preferred Reporting Items for Systematic Reviews and Meta-Analysis (PRISMA) guidelines [10] and followed the recommendations of the Cochrane Collaboration Handbook for Systematic Reviews of Interventions [11]. The protocol was registered in the International Prospective Register of Systematic Reviews (PROSPERO) with the following registration ID: 1019642.

### 2.2. Eligibility Criteria

We included studies that met the following eligibility criteria: (1) RCTs; (2) that compared PF-DESs with BP-DESs; and (3) in adult patients with CAD undergoing PCI. A cutoff of <100 µm for strut thickness was selected as an inclusion criterion for the BP-DESs arm, aiming to more effectively isolate and evaluate the clinical impact of polymer characteristics. No such restriction was applied to polymer-free DESs. We excluded studies with non-randomized design if the comparator arm used biodegradable polymer DESs with strut thickness > 100 μm or permanent polymer DESs, involving peripheral angioplasty or with overlapping populations were also excluded. Trials conducted in both acute coronary syndrome (ACS) and chronic coronary syndrome (CCS) settings were considered eligible; those conducted in other clinical contexts were excluded. The primary outcome of this meta-analysis was 12-month target lesion revascularization (TLR), selected based on its clinical relevance, consistency across studies, and established role as a key measure of device performance. Secondary outcomes included myocardial infarction (MI), cardiac death, all-cause death, stent thrombosis, and angiographic- and OCT-derived endpoints.

### 2.3. Information Sources and Search Strategy

A comprehensive search was conducted in PubMed, The Cochrane Central Register of Controlled Trials (CENTRAL), and Embase (via Ovid) from inception to 1 February 2025. No restrictions were applied regarding publication type or language. Additional references were identified by manually screening the bibliographies of included studies and through backward and forward citation tracking. The complete search strategy for Pubmed is reported in Supplementary Materials Table S1.

### 2.4. Study Selection

Two reviewers (M.M. and S.S.) independently screened titles and abstracts to assess eligibility. Full texts of potentially relevant studies were then retrieved and assessed independently. Discrepancies were resolved through discussion and consensus. The selection process was documented in sufficient detail to allow for the construction of a PRISMA 2020 flow diagram.

### 2.5. Data Collection

A standardized data extraction form, piloted on an initial sample of three studies, was used to collect relevant information, including study characteristics, outcomes, and risk of bias assessments. Data extraction was performed independently by two authors (M.M. and S.S.).

### 2.6. Data Items

We extracted data on all predefined primary and secondary outcomes, as well as on study-level characteristics such as population demographics, clinical setting, stent type, and comparator specifications. No assumptions were made for missing or unclear data, and study authors were not contacted for additional information.

### 2.7. Risk of Bias Assessment

The risk of bias in the included studies was independently assessed by two reviewers (M.M. and S.S.) using the Cochrane Risk of Bias 2 (RoB 2) tool [12]. Disagreements were resolved by consensus.

### 2.8. Data Synthesis and Statistical Analysis

Quantitative synthesis was performed using Review Manager (RevMan) version 5, applying a random-effects model for all meta-analyses. For dichotomous outcomes, results were expressed as risk ratios (RRs) with corresponding 95% confidence intervals (CIs). Continuous outcomes were reported as mean differences (MDs) with 95% CIs. Statistical heterogeneity across studies was assessed using the I^2^ statistic. Heterogeneity was considered significant when I^2^ resulted in >50%. We did not impute missing data or conduct statistical conversions of outcome measures.

### 2.9. Reporting Bias

Potential publication bias and small-study effects were assessed through visual inspection of funnel plots.

### 2.10. Additional Analyses

Subgroup and sensitivity analyses were performed for outcomes that exhibited substantial heterogeneity (I^2^ > 50%), specifically 12-month TLR and 12-month cardiac death. For TLR, two exploratory analyses were conducted. First, a prespecified subgroup analysis compared studies evaluating the BioFreedom stent versus those using other polymer-free DESs. Summary estimates were calculated separately for each subgroup to investigate potential differences in treatment effect based on stent technology. Second, a sensitivity analysis was performed excluding Hansen 2022 [13]—the only study conducted exclusively in an emergent PCI setting (primary PCI for STEMI)—to assess whether clinical context influenced the pooled estimate or heterogeneity. For cardiac death, a similar subgroup analysis by device type (BioFreedom vs. non-BioFreedom) was conducted. Additionally, a sensitivity analysis excluding Hansen 2022 [13] was carried out to determine the influence of emergent PCI on heterogeneity and effect estimates. To address potential surveillance bias, an additional sensitivity analysis for TLR was conducted excluding ISAR-TEST 3, the only study among those reporting TLR that mandated systematic angiographic follow-up regardless of clinical indication. In such trials, revascularization decisions may be influenced by the detection of angiographic restenosis in asymptomatic patients, potentially leading to an overestimation of TLR rates. No formal subgroup or sensitivity analyses were performed for angiographic and OCT outcomes due to the limited number of contributing studies and substantial methodological variability across trials.

### 2.11. Certainty of Evidence

The certainty of evidence for all key clinical, angiographic, and OCT outcomes was assessed using the GRADE approach with the support of GRADEpro GDT software (McMaster University, 2023, accessed May 2025). Five domains were considered: risk of bias, inconsistency, indirectness, imprecision, and publication bias. For each outcome, judgments were based on the number and quality of included studies, I^2^ values, confidence interval widths, outcome type (clinical vs. surrogate), and applicability to clinical practice.

## 3. Results

### 3.1. Study Selection

**Figure 1:**
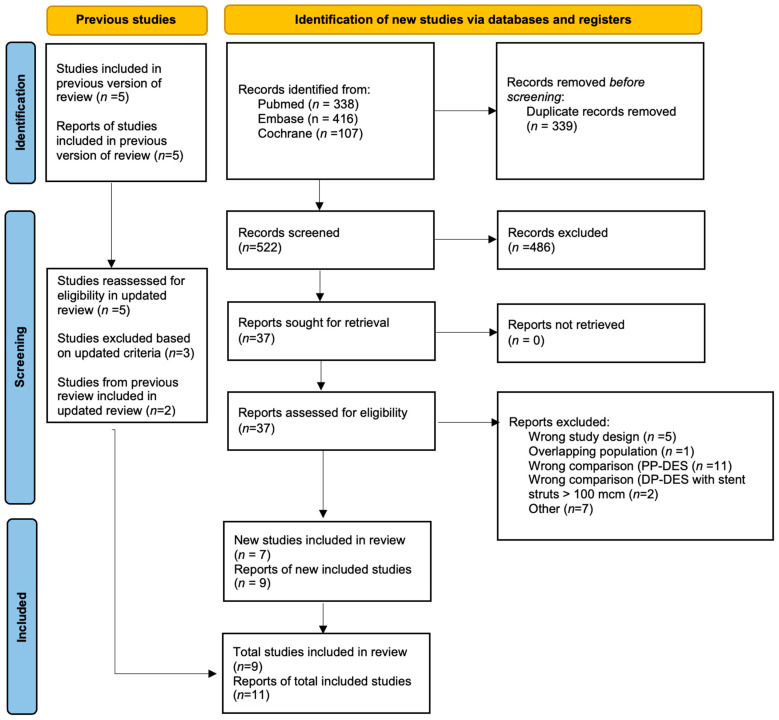
PRISMA 2020 flow diagram for updated systematic reviews, which included searches of databases and registers only.

A total of 861 records were identified through database searches (PubMed = 338, Embase = 416, Cochrane = 107). After the removal of 339 duplicates, 522 records were screened based on titles and abstracts. Of these, 37 full-text articles were assessed for eligibility. Twenty-eight studies were excluded for the following reasons: wrong study design (*n* = 5), overlapping population (*n* = 1), inappropriate comparator (PP-DES, *n* = 11; DP-DES with strut thickness > 100 μm, *n* = 2), or other reasons (*n* = 7). Nine studies were ultimately included in the final qualitative and quantitative synthesis, for a total of eleven reports: ISAR-TEST 3 [14,15], SORT-OUT IX [16,17], Hansen et al., 2022 [13], Gomez et al., 2021 (FUNCOMBO) [18], Hong et al., 2021 [19], Otaegui et al., 2022 (FRIENDLY-OCT) [20], Piccolo et al., 2025 [21], Tao et al., 2021 (RECOVERY) [22], and Viswanathan et al., 2018 [23]. Two reports (Mehilli et al., 2008 [14], Byrne et al., 2009 [15]) both referred to the ISAR-TEST 3 trial, and two (Jensen et al., 2020 [16], Gregersen et al., 2022 [17]) reported complementary data from the SORT-OUT IX trial. The study selection process is detailed in the PRISMA flowchart (Figure 1).

### 3.2. Study Characteristics

The studies were conducted across multiple countries—Germany, Denmark, Spain, South Korea, Italy, China, and India—encompassing a total of 9597 patients and 11,454 lesions. Sample sizes ranged from 60 to 3151 patients. The study populations included were varied in terms of clinical presentation and enrollment criteria. Four studies—SORT-OUT IX [16,17], Piccolo et al., 2025 [21], Tao et al., 2021 [22], and Viswanathan et al., 2018 [23]—adopted an “all-comers” PCI strategy, allowing for broad inclusion criteria with minimal exclusions. Among these, Viswanathan et al., 2018 [23] was the only trial limited to patients undergoing purely elective procedures. Two studies (Hansen et al., 2022 [13], Gomez et al., 2021 [18]) focused specifically on patients with ST-elevation myocardial infarction (STEMI) or other high-risk ACS. Hong et al., 2021 [19] and ISAR-TEST 3 trials exclusively enrolled patients undergoing non-emergent PCI, excluding those with acute myocardial infarction, cardiogenic shock, or other high-risk features. Similarly, Otaegui et al., 2022 [20] limited inclusion to stable patients undergoing non-emergent PCI, with intra-patient randomization and 3-month OCT follow-up. Devices included BioFreedom, Coroflex ISAR, Cre8 EVO, Nano, and others, among PF-DESs and Orsiro, Ultimaster, SYNERGY, Combo, and others, among BP-DESs. Strut thickness of PF-DESs ranged from 60 to 140 µm, while BP-DESs varied from 60 to 100 µm. Comprehensive details of baseline study characteristics are provided in Table 1, Table 2 and Table 3. Available data on dual antiplatelet therapy (DAPT) regimen and duration are summarized in Table 4. Most studies reported standard use of aspirin and clopidogrel, with durations ranging from 1 to 12 months depending on trial design. However, two trials (Gomez et al., 2021 [18] and Tao et al., 2021 [22]) did not specify DAPT regimens, and only a few studies provided stratified data on the P2Y_12_ inhibitor type, limiting further comparative analysis.

### 3.3. 12-Month Clinical Outcomes

**Figure 2:**
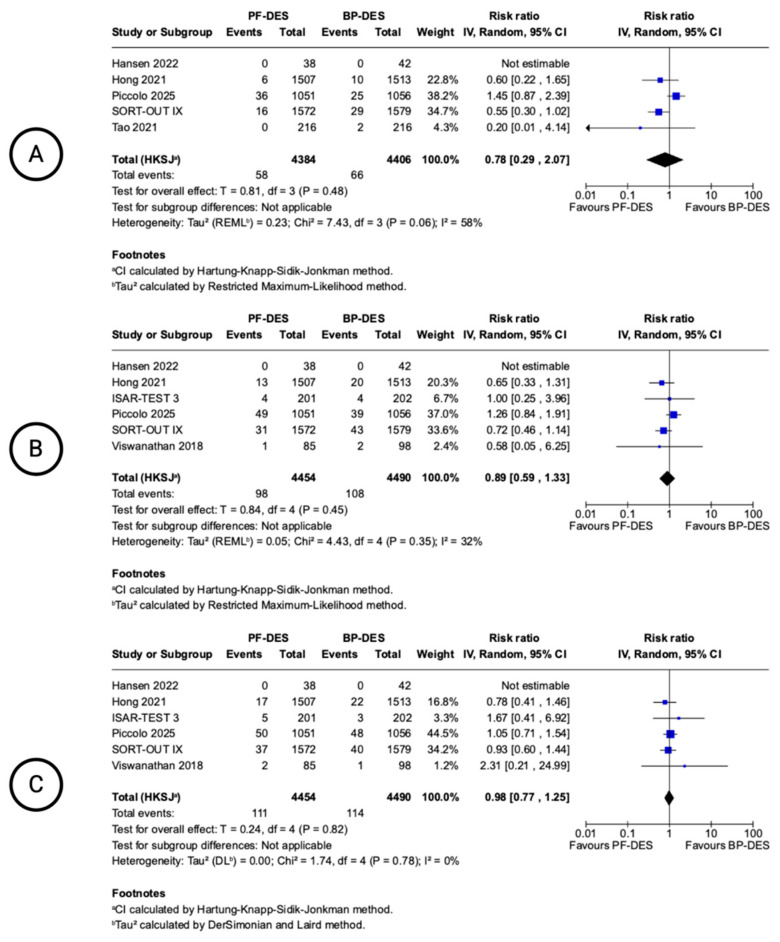
Forest plots showing pooled incidence of 12-month clinical outcomes. Cardiac death (**A**), all-cause mortality (**B**), myocardial infarction (**C**). Included Hansen 2022 [13], Hong 2021 [19], Piccolo 2025 [21], SORT-OUT IX trial [16,17], Tao 2021 [22], ISAR-TEST 3 trial [14,15], Viswanathan 2018 [23], Hansen 2022 [13].

Figure 2 presents the forest plots for the pooled incidence of 12-month clinical outcomes, including cardiac death (panel A), all-cause mortality (panel B), and MI (panel C). For cardiac death, the pooled analysis from five studies showed a non-significant reduction in risk with PF-DESs compared to BP-DESs (RR 0.78, 95% CI 0.29–2.07; I^2^ = 58%), with moderate heterogeneity. Regarding all-cause mortality (panel B), the pooled risk ratio was 0.89 (95% CI 0.59–1.33; I^2^ = 32%), with no statistically significant difference between PF-DESs and BP-DESs and low heterogeneity. For MI (panel C), the analysis included six studies and showed a pooled RR of 0.98 (95% CI 0.77–1.25; I^2^ = 0%), indicating no difference in 12-month MI rates between groups and no evidence of heterogeneity.

### 3.4. 24-Month Clinical Outcomes

**Figure 3:**
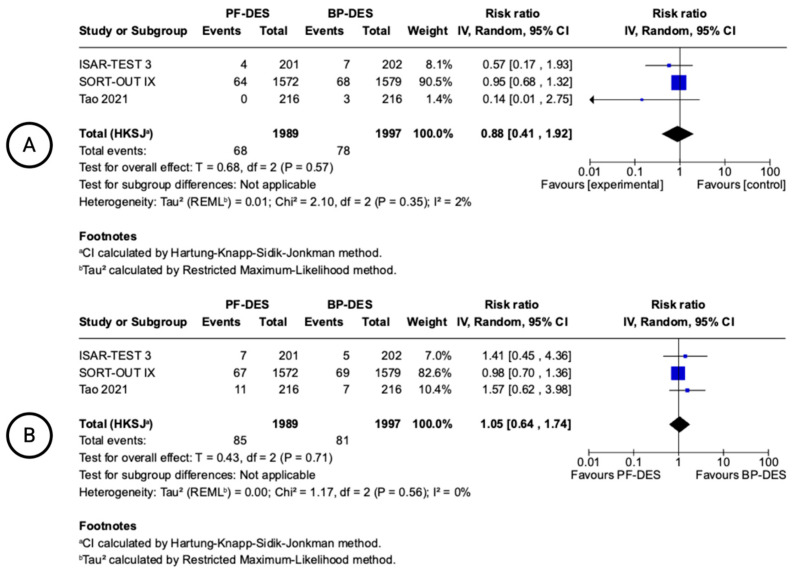
Forest plots showing pooled incidence of 24-month clinical outcomes. All-cause death (**A**), myocardial infarction (**B**). Included ISAR-TEST 3 trial [14,15], SORT-OUT IX trial [16,17], Tao 2021 [22].

Three studies reported MI and all-cause mortality at 24 months (Figure 3). The pooled RR for MI was 1.05 (95% CI: 0.64–1.74; I^2^ = 0%), and for all-cause death, it was 0.88 (95% CI: 0.41–1.92; I^2^ = 2%). These findings indicate no statistically significant differences and minimal heterogeneity.

### 3.5. 12-Month Device-Oriented Clinical Outcomes

**Figure 4:**
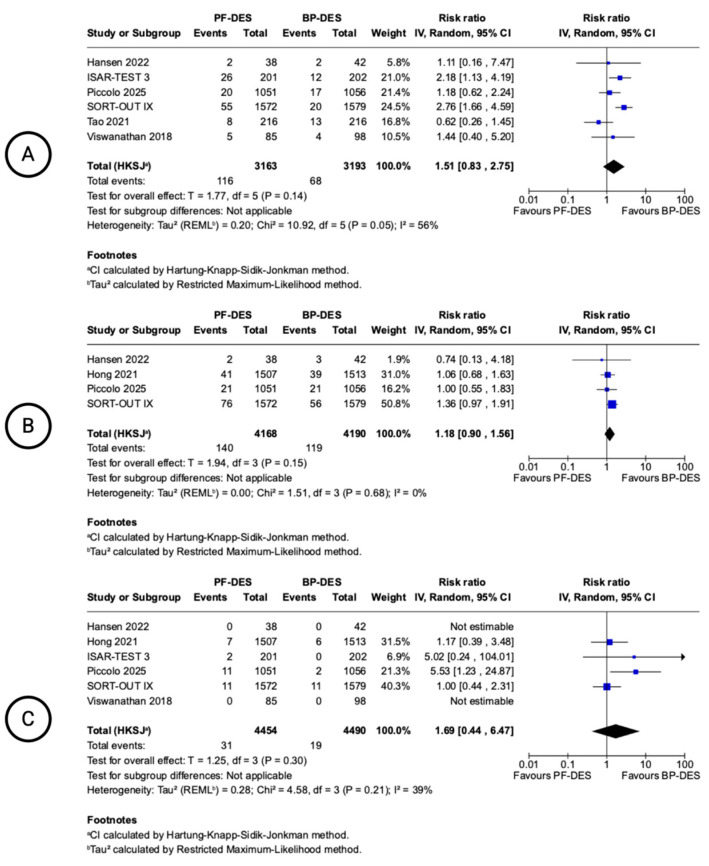
Forest plots showing pooled incidence of 12-month clinical-oriented device outcomes. Target lesion revascularization (**A**), target vessel revascularization (**B**), stent thrombosis (**C**). Included Hansen 2022 [13], ISAR-TEST 3 trial [14,15], Piccolo 2025 [21], SORT-OUT IX trial [16,17], Tao 2021 [22], Viswanathan 2018 [23], Hong 2021 [19].

TLR was reported in six studies (Figure 4). The pooled RR was 1.51 (95% CI: 0.83–2.75), indicating no statistically significant difference between PF-DESs and BP-DESs. A trend favoring BP-DESs was noted, with moderate heterogeneity (I^2^ = 42%). Four studies reported TVR. The pooled RR was 1.18 (95% CI: 0.90–1.56; I^2^ = 10%), showing no statistically significant difference and low heterogeneity. Definite or probable ST was reported in six studies. The pooled RR was 1.69 (95% CI: 0.44–6.47), with no statistically significant difference between groups and moderate heterogeneity (I^2^ = 39%).

### 3.6. Angiographic and OCT Outcomes

**Figure 5:**
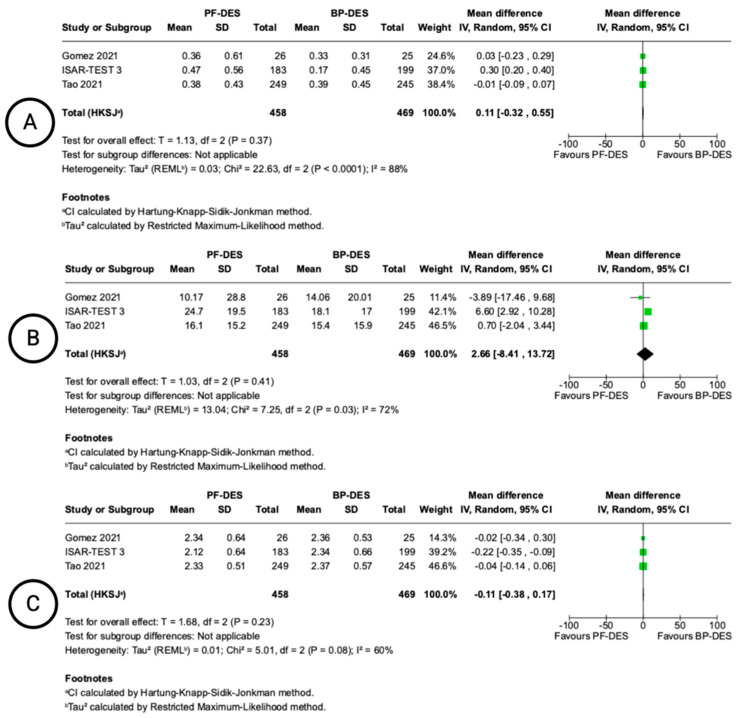
Forest plots showing pooled incidence of angiographic outcomes. In–stent late lumen loss (**A**), in–stent binary restenosis (**B**), in–stent minimal lumen diameter (**C**). Included Gomez 2021 [18], ISAR-TEST 3 [14,15], Tao 2021 [22].

Angiographic outcomes were reported in three studies, with follow-up ranging from 6 to 9 months (Figure 5). No statistically significant differences were observed between PF-DESs and BP-DESs in in-stent LLL with a MD of 0.11 mm (−0.32 to 0.55 95% CI; I^2^ = 88%), binary restenosis (MD: 2.66%; −8.41 to 13.72 95% CI; I^2^ = 88%), or MLD (MD: −0.11 mm; −0.38 to 0.17 95% CI; I^2^ = 60%). Heterogeneity was considerable for all angiographic endpoints.

**Figure 6:**
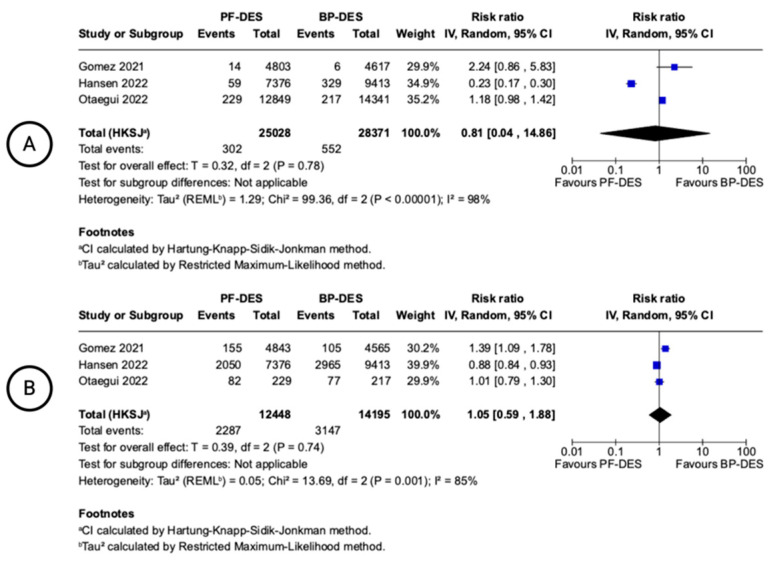
Forest plots showing pooled incidence of OCT outcomes. Number of malapposed struts (**A**), number of uncovered struts (**B**). Gomez 2021 [18], Hansen 2022 [13], Otaegui 2022 [20].

OCT-derived outcomes were available from three studies, with follow-up times ranging from 1 to 6 months (Figure 6). No statistically significant differences were observed between PF-DESs and BP-DESs in the risk of malapposed struts (RR: 0.81; 0.04 to 14.86 95% CI; I^2^ = 98%) or uncovered struts (RR: 1.05; 0.59 to 1.88 95% CI; I^2^ = 85%). Heterogeneity was substantial for both outcomes.

Given that only three studies contributed data to each angiographic and OCT outcome, statistical exploration of heterogeneity through meta-regression or subgroup analysis was not feasible. These results are therefore reported descriptively and discussed narratively.

### 3.7. Additional Analyses

To explore potential sources of heterogeneity in outcomes with I^2^ > 50%, we conducted several predefined sensitivity and subgroup analyses for TLR and cardiac death. Additional analyses are reported in Supplementary Materials Figures S1–S5. For TLR, the exclusion of Hansen 2022 [13]—the only trial enrolling exclusively patients undergoing emergent PCI—resulted in a pooled RR of 1.53 (0.73–3.20 95% CI), with increased heterogeneity (I^2^ = 63%), suggesting that clinical setting alone did not account for the observed variability. In a device-based subgroup analysis, studies evaluating the BioFreedom stent showed a numerically higher, though non-significant, risk of TLR with PF-DESs (RR: 2.60; 0.15–46.59 95% CI; I^2^ = 0%), whereas no significant effect was observed in the non-BioFreedom subgroup (RR: 1.26; 0.53–2.97 95% CI; I^2^ = 47%). To account for potential surveillance bias, we conducted an additional sensitivity analysis excluding ISAR-TEST 3—the only trial with protocol-mandated angiographic follow-up among those reporting TLR—which yielded a pooled RR of 1.35 (0.64–2.87 95% CI; I^2^ = 60%). For cardiac death, the exclusion of Hansen et al., 2022 did not materially alter the effect estimate (RR: 0.78; 0.29–2.07 95% CI) or reduce heterogeneity (I^2^ = 58%). However, in the subgroup analysis based on the stent platform, studies evaluating BioFreedom demonstrated a statistically significant reduction in cardiac death with polymer-free DESs (RR: 0.57; 0.35–0.90 95% CI; I^2^ = 0%), whereas no such effect was observed in the non-BioFreedom subgroup (RR: 0.97; 0.00–23,942.44 95% CI; I^2^ = 37%).

### 3.8. Quality Assessment and Publication Bias

All studies were judged to have an overall low risk of bias, except for SORT-OUT IX, which was rated as having some concerns due to deviations from intended interventions, and Viswanathan 2018, which had some concerns related to outcome measurement. No study was rated at high risk of bias. A detailed summary of the domain-level risk of bias assessments is provided in Supplementary Materials Table S2. Given the limited number of studies available for each outcome, formal statistical tests for publication bias were not performed. Visual inspection of funnel plots did not reveal clear asymmetry. However, the interpretation of funnel plots is limited when few studies are included, and no firm conclusions regarding the presence or absence of publication bias can be drawn. All funnel plots are provided in the Supplementary Materials Figures S6–S18.

### 3.9. Certainty of Evidence

The certainty of evidence was rated as high for myocardial infarction and moderate for target lesion revascularization and stent thrombosis. Cardiac death and all-cause death were rated as low-certainty due to inconsistency and imprecision. All angiographic and OCT endpoints were rated as very low certainty due to very serious inconsistency, indirectness, and imprecision. The full GRADE ratings are summarized in Table 5 (clinical outcomes) and Table 6 (angiographic and OCT outcomes).

## 4. Discussion

This systematic review and meta-analysis provides an updated synthesis of randomized evidence comparing PF-DESs and BP-DESs in patients undergoing PCI. Overall, no statistically significant differences were observed between PF-DESs and BP-DESs in terms of myocardial infarction, all-cause death, or stent thrombosis at 12 or 24 months. Although not reaching statistical significance, a trend favoring BP-DESs was observed in TLR, whereas a non-significant trend favoring PF-DESs was noted for cardiac death. Pooled analyses of angiographic and OCT endpoints revealed no statistically significant differences between PF-DESs and BP-DESs.

Importantly, in a predefined subgroup analysis, a statistically significant reduction in cardiac death was observed with the BioFreedom stent; however, this result, derived from a limited number of trials, should be considered hypothesis-generating and interpreted cautiously. This signal was not observed among non-BioFreedom polymer-free platforms, suggesting that the benefit may be device-specific rather than representative of the entire PF-DES class. These findings warrant further investigation in trials explicitly powered for hard endpoints and stratified by a stent platform. While subgroup analysis was performed for the BioFreedom stent given its evaluation in multiple RCTs, an equivalent stratification by individual BP-DES devices was not feasible due to heterogeneity and limited representation. The BP-DES group included diverse platforms such as Orsiro, Ultimaster, Synergy, COMBO, and EES, each tested in only one or two studies. This diversity in strut thickness, polymer degradation kinetics, and drug release profiles makes device-level conclusions for BP-DESs less robust. Therefore, BP-DESs were grouped based on their shared class characteristics (biodegradable polymer and drug elution) rather than the device brand. Conversely, the device-based subgroup analysis for TLR did not reveal statistically significant differences between BioFreedom and non-BioFreedom subgroups, although heterogeneity was confined to the latter. This observation suggests that technical features such as strut thickness, drug delivery kinetics, or reservoir-based technologies may influence restenosis-related outcomes more than the presence or absence of a polymer alone.

To explore potential drivers of heterogeneity, several sensitivity analyses were conducted. The exclusion of Hansen et al., 2022 [13], the only trial enrolling exclusively patients with STEMI, did not substantially affect effect estimates or reduce heterogeneity for either TLR or cardiac death, indicating that the emergent PCI setting was unlikely the main source of variability. Similarly, the exclusion of ISAR-TEST 3, the only study with protocol-mandated systematic angiographic follow-up among those reporting TLR, did not eliminate heterogeneity. This trial was also the only one to mandate protocol-driven angiographic follow-up, which may have led to higher TLR detection rates through systematic surveillance. In contrast, the remaining trials performed clinically indicated angiography only, potentially underestimating restenosis-related reinterventions. This difference in outcome ascertainment likely introduced detection bias and should be considered when interpreting TLR results. These findings support the notion that observed variability likely stems from multiple interacting factors, including stent platform characteristics, patient selection, and outcome ascertainment methods.

The findings of this meta-analysis are broadly consistent with the individual results of the randomized controlled trials included. For TLR, most studies reported no statistically significant difference, although numerically higher event rates were observed with PF-DESs in ISAR-TEST 3, SORT-OUT IX, and RECOVERY, in line with the direction of the pooled estimate. SORT-OUT IX notably found a significantly higher TLR rate with BioFreedom, largely driven by events in the first year. In contrast, for cardiac death, the subgroup analysis revealed a significant reduction in the BioFreedom group that was not detected in individual studies, likely due to limited power. This finding highlights the utility of meta-analytic approaches in detecting potentially meaningful signals. Angiographic and OCT outcomes were also consistent with trial-level data: studies such as FUNCOMBO and FRIENDLY-OCT showed small but inconsistent differences in in-stent restenosis and strut healing parameters, favoring BP-DESs in most but not all metrics. In contrast, the GRADE assessment rated the certainty of evidence as high for myocardial infarction and moderate for target lesion revascularization and stent thrombosis, lending strength to the reliability of the clinical findings reported.

Differences in stent platform design may mechanistically influence vascular response and clinical outcomes. Polymer-free DESs eliminate the polymeric carrier, potentially reducing chronic inflammation, hypersensitivity reactions, and delayed endothelial healing. In contrast, biodegradable polymer DESs aim to minimize these issues by allowing polymer resorption, but still expose the vessel wall to polymers during the initial healing phase. Strut thickness is another critical determinant: thinner struts reduce flow disturbance, promote more rapid endothelial coverage, and are associated with lower neointimal proliferation. Moreover, elution profiles differ between platforms—BioFreedom releases drugs rapidly without sustained concentration, while BP-DESs generally allow for more prolonged and controlled drug delivery. These mechanistic differences may help explain the subtle variations observed in angiographic outcomes and device-related endpoints.

Interpretation of safety outcomes, such as stent thrombosis, may also be influenced by differences in DAPT duration and intensity across studies. Although all included trials mandated dual antiplatelet therapy, the planned duration varied from 1 to 12 months, and the choice of P2Y_12_ inhibitor was not uniformly reported. These inconsistencies, summarized in Table 4, limit the ability to draw firm conclusions regarding the interaction between the stent platform and antiplatelet regimen and represent a source of residual confounding that should be acknowledged.

Substantial heterogeneity was observed across angiographic- and OCT-derived outcomes, which limits the interpretability of pooled estimates. For angiographic endpoints such as late lumen loss and binary restenosis, differences in follow-up timing, image acquisition protocols, and the use of systematic versus clinically driven angiography likely contributed to variability. Similarly, the limited number of OCT trials, variability in follow-up time (1 to 6 months), and use of different definitions for strut coverage and malapposition complicated the synthesis of morphologic endpoints. These results should be interpreted descriptively and considered hypothesis-generating rather than definitive. These findings are further supported by our GRADE assessment, which confirmed that the certainty of evidence for all angiographic and OCT outcomes was very low. This reinforces the hypothesis-generating nature of these endpoints and highlights the need for further dedicated imaging trials with standardized protocols and longer follow-ups.

Although bleeding, stroke, and rehospitalization are relevant for assessing net clinical benefit, these outcomes were not consistently or uniformly reported across the included studies. For example, the One-month DAPT trial reported major bleeding rates of 1.7% versus 2.5% (PF-DES vs. BP-DES; *p* = 0.136), and Viswanathan et al. reported stroke in 2.04% of BP-DES patients and 0% in PF-DES. Rehospitalization data were indirectly available in some studies, such as SORT-OUT IX and Viswanathan et al., but were not stratified or detailed. Due to these inconsistencies, no formal meta-analysis was performed, but available findings are discussed qualitatively to inform clinical interpretation.

This meta-analysis has several limitations. First, information on the duration and type of DAPT was not consistently reported across studies, precluding analysis of its impact on ischemic and bleeding outcomes. Second, despite efforts to restrict heterogeneity by selecting trials comparing PF-DESs and BP-DESs with thin or ultrathin struts, variability in device platforms and clinical settings may still have influenced the results. Third, formal testing for publication bias was not feasible due to the small number of studies per outcome. Additionally, some subgroup and sensitivity analyses were exploratory in nature and should be interpreted with caution. Furthermore, angiographic and OCT outcomes were reported by only three studies each, preventing formal exploration of heterogeneity. Consequently, pooled estimates for these endpoints should be interpreted cautiously and considered to be hypothesis-generating. To support interpretation, we performed a GRADE assessment of the certainty of evidence for angiographic and OCT outcomes. All were graded as very low certainty, due to serious or very serious concerns related to inconsistency, indirectness, and imprecision. These ratings, now summarized in Table 5, reinforce the hypothesis-generating nature of these findings and the need for further trials specifically powered for morphological endpoints. Consequently, pooled estimates for these endpoints should be interpreted cautiously and considered hypothesis-generating. Moreover, the GRADE assessment confirmed very low certainty of evidence for all angiographic and OCT outcomes, further limiting the strength of inference. Moreover, the GRADE assessment confirmed that the certainty of evidence was very low for angiographic and OCT outcomes, and low for some clinical endpoints such as cardiac and all-cause death. This further limits the confidence in the pooled estimates for these outcomes. Additionally, systematic angiographic follow-up was mandated only in the ISAR-TEST 3 trial, while all other studies relied on clinical indications. This inconsistency in surveillance strategies may have influenced the detection of restenosis and the subsequent need for TLR, representing a potential source of detection bias.

An important methodological consideration of our meta-analysis is the selective application of the <100 µm strut thickness criterion exclusively to biodegradable polymer DESs, while no such restriction was applied to polymer-free devices. The decision to apply the <100 µm strut thickness criterion exclusively to biodegradable polymer DESs was based on both mechanistic rationale and supporting evidence. In polymer-based platforms, strut thickness plays a critical role in determining vascular healing, restenosis, and thrombosis risk. Randomized and observational studies have demonstrated that thinner-strut stents are independently associated with lower rates of both angiographic and clinical restenosis compared to thicker-strut devices, particularly in vessels >2.75 mm in diameter [6,7]. Furthermore, contemporary reviews confirm that thinner struts reduce neointimal hyperplasia, promote endothelialization, and improve overall stent performance when a polymer is present [5]. These considerations support our approach of applying the strut thickness criterion selectively in the BP-DES group while allowing broader inclusion of PF-DESs, which lack the polymer-related inflammatory stimulus.

In conclusion, this meta-analysis suggests that PF-DESs and BP-DESs have comparable safety and efficacy profiles in the general population undergoing PCI. However, the finding of a significantly lower risk of cardiac death with the BioFreedom stent in subgroup analysis may reflect device-specific advantages and warrants further study. Future trials comparing PF-DES platforms head-to-head are needed to clarify whether polymer-free technologies offer clinical advantages over contemporary biodegradable polymer DESs.

## Figures and Tables

**Table 1:** Baseline characteristics of included studies.

Study	Design	Country	N. Patients PF/BP	N. Lesions Treated PF/BP	PF-DES, Name	PF-DES, Platform Material	PF-DES, Strut Thickness (mcm)	PF-DES, Drug Eluted	BP-DES, Name	BP-DES, Platform Material	BP-DES, Strut Thickness (mcm)	BP-DES, Drug Eluted	BP-DES, Polymer
ISAR-TEST 3 [14,15]	RCT	Germany	201/202	231/232	NA	316L stainless steel	140	Rapamycine	NA	316L stainless steel	87	Sirolimus	PLA
SORT-OUT IX [16,17]	RCT	Denmark	1572/1579	1966/1985	BioFreedom	316L stainless steel	114–119	Biolimus A9	Orsiro	CoCr	60–80	Sirolimus	PLLA
Hansen 2022 [13]	RCT	Denmark	38/42	38/42	BioFreedom	316L stainless steel	120	Biolimus A9	Orsiro	CoCr	60–80	Sirolimus	PLLA
Gomez 2021 [18]	RCT	Spain	30/30	29/31	BioFreedom	316L stainless steel	120	Biolimus A9	Combo	CoCr	100	Sirolimus	PLLA
Hong 2021 [19]	RCT	South Korea	1507/1513	1507/1513	BioFreedom	316L stainless steel	120	Biolimus A9	Ultimaster	CoCr	80	Sirolimus	PLLA
Otaegui 2022 [20]	RCT	Spain	70/70	70/70	Coroflex ISAR	CoCr	60	Probucol	Ultimaster	CoCr	80	Sirolimus	PLLA
Piccolo 2025 [21]	RCT	Italy	1051/1056	1513/1529	Cre8 EVO	CoCr	70–80	Amphilimus	SYNERGY	PtCr	74–81	Everolimus	PLGA
Tao 2021 [22]	RCT	China	216/216	249/245	Nano	CoCr	90–100	Sirolimus	Combo	CoCr	100	Sirolimus	PLLA
Viswanathan 2018 [23]	RCT	India	91/113	91/113	NA	316L stainless steel	87	Sirolimus	NA	316L stainless steel	87	Sirolimus	PDLLA

BP: biodegradable polymer; CoCr: cobaltum–chromium; DES: drug-eluting stent; NA: not available; PDLLA: poly (d,l) lactic acid; PLA: polylactic acid; PLGA: poly d,l lactide co-glycolide; PLLA: poly-L-lactide; PF: polymer-free; PtCr: platinum–chromium; RCT: randomized controlled trial.

**Table 2:** Clinical baseline characteristics of included studies.

Study	Clinical Setting	Age ^†^, y PF/BP	BMI ^†^, PF/BP	Male, % PF/BP	DM, % PF/BP	HTN, % PF/BP	Smokers, % PF/BP	Dyslipidemia, % PF/BP	Prior MI, % PF/BP	Prior CABG, % PF/BP	Prior PCI, % PF/BP	LVEF ^†^, % PF/BP
ISAR-TEST 3 [14,15]	Non-emergent PCI ^1^	66.8/66.5	27.2/27.4	78.1/78.2	27.2/28.7	67.2/71.8	17.8/16.3	71.1/71.3	32.9/32.2	13.4/10.4	NA	53.5/53.8
SORT-OUT IX [16,17]	All comers	66.4/66.1	27.8/27.6	77.5/77.3	19.3/19.2	59.0/56.0	29.8/29.3	55.0/51.5	14.7/15.2	8.4/7.0	20.9/20.9	NA
Hansen 2022 [13]	STEMI	61.3/62.6	27.3/27.1	81.6/83.3	5.3/11.9	34.2/40.5	45.2/39.5	18.4/14.2	7.9/2.4	0/0	7.9/4.8	NA
Gomez 2021 [18]	STEMI	57.1/57.2	28.1/27.4	93.1/77.4	20.7/6.5	48.3/32.3	69.0/67.7	58.6/51.6	NA	NA	0/3.2	52.0/52.4
Hong 2021 [19]	Non-emergent PCI ^2^	67.0/67.0	24.7/24.7	69/69	37.0/38.0	67.0/66.0	17.0/16.0	81.0/82.0	4.0/4.0	1.0/2.0	16/18.1	63.0/63.0
Otaegui 2022 [20] ^§^	Non-emergent PCI ^3^	67.0/67.0	NA	78.6	30	75.7	55.7	61.4	17.1	4.3	21.4	NA
Piccolo 2025 [21]	All comers	63.8/64.0	28.0/28.2	77.2/78.7	30.6/31.8	73.9/75.7	49.9/49.8	62.4/62.5	20.5/19.3	3.4/4.2	21.3/21.8	49.2/49.1
Tao 2021 [22]	All comers	59.3/58.3	NA	63.4/68.1	21.3/19.9	60.2/53.7	43.1/44.9	17.1/12.5	13.0/13.4	0.5/0.5	9.3/9.7	60.2/60.0
Viswanathan 2018 [23]	Elective PCI	55.8/56.9	NA	87.9/73.4	49.4/44.2	49.2/51.3	NA	NA	NA	NA	NA	57.3/58.7

^§^ intra-patient randomized study; ^†^ mean or median; BMI: body mass index; BP: biodegradable polymer; CABG: coronary artery bypass grafting; DM: diabetes mellitus; HTN: arterial hypertension; LVEF: left ventricular ejection fraction; MI: myocardial infarction; NA: not available; PCI: percutaneous coronary intervention; PF: polymer-free; STEMI: ST-elevated myocardial infarction. ^1^ Patients with target lesion located in Left Main Stem or in a by-pass, in-stent restenosis, acute myocardial infarction, cardiogenic shock, malignancy, or life expectancy < 12 months were excluded. ^2^ Acute myocardial infarction, complex morphologies (such as aorto-ostial, unprotected Left Main, chronic total occlusions, graft thrombosis, heavily calcified lesions) were excluded. ^3^ Unstable conditions, such as STEMI or cardiogenic shock, complex scenarios prone to stent malapposition (bifurcation needing double stenting, severe calcification, coronary ectasia, thrombus, or severe angulation) were excluded.

**Table 3:** Angiographic and procedural baseline characteristics of included studies.

Study	TL:LAD, %, PF/BP	TL:LCX, %, PF/BP	TL:RCA, %, PF/BP	TL:LM, %, PF/BP	Stent Diameter ^†^, mm, PF/BP	Stent Length ^†^, mm, PF/BP	Diameter Stenosis ^†^, %, PF/BP	MLD, pre, mm, PF/BP	Predilatation, %, PF/BP	Postdilatation, %, PF/BP
ISAR-TEST 3 [14,15]	39.8/46.0	29.5/22.2	30.7/31.8	NA	2.75/2.74	14.3/13.9	58.8/61.5	1.13/1.06	NA	NA
SORT-OUT IX [16,17]	43.0/43.0	23.0/22.4	30.0/31.3	2.5/2.9	NA	30.6/31.1	NA	NA	91.8/90.2	NA
Hansen 2022 [13]	42.9/31.6	11.9/13.2	45.2/55.3	NA	NA	25.0/25.6	NA	NA	NA	NA
Gomez 2021 [18]	44.8/48.4	20.7/16.1	34.5/35.5	NA	3.08/3.04	20.1/18.3	NA	2.70/2.69	20.6/22.5	3.44/12.9
Hong 2021 [19]	56.0/55.0	19.0/18.1	25.0/26.7	NA	3.1/3.1	20.3/20.5	71.4/71.4	0.8/0.8	NA	NA
Otaegui 2022 ^§^ [20]	34.3/34.3	35.7/31.4	30.0/34.3	NA	2.9/3.0	19.9/19.4	71.4/66.3	0.88/1.02	71.4/70	38.6/47.1
Piccolo 2025 [21]	46.3/44.6	22.9/23.3	29.5/29.6	1.3/2.4	3.0/3.0	29.1/29.1	NA	NA	62.6/60.9	61.9/57.2
Tao 2021 [22]	47.0/51.8	24.1/19.2	28.9/29.0	NA	3.17/3.17	24.9/24.1	68.4/67.7	0.92/0.94	91.6/91.8	72.3/74.3
Viswanathan 2018 [23]	NA	NA	NA	NA	3.18/2.97	28.2/29.6	NA	NA	68.9/67.9	26.0/38.3

^§^ intra-patient randomized study; ^†^ mean or median; BP: biodegradable polymer; LAD: left anterior descendant; LCX: left circumflex: LM: Left Main; MLD: minimal lumen diameter; NA: not available; PF: polymer-free; TL: target lesion.

**Table 4:** Summary of antiplatelet therapy regimens across included studies.

Study	DAPT Duration PF-DES	DAPT Duration BP-DES	ASA (%)	Clopidogrel (%)	Prasugrel (%)	Ticagrelor (%)	Notes
ISAR-TEST 3 [14,15]	12 months	12 months	All patients	All patients	Not used	Not used	Clopidogrel 600 mg pre-PCI, then 75 mg/day × 12 m
SORT-OUT IX [16,17]	12 months	12 months	All patients	Standard per protocol	Allowed	Allowed	DAPT per guideline, no stratified data
Hansen 2022 [13]	1 month	12 months	All patients	All patients	Not reported	Not reported	DAPT: ASA + Clopidogrel for 1 month (PF), 12 months (BP)
Hong 2021 [19]	1 month	6–12 months	All patients	95.3	0.4	4.3	Data from Table 2 of publication
Otaegui 2022 [20]	3 months	3 months	All patients	All patients	Not used	Not used	3 months DAPT confirmed in Methods Section.
Piccolo 2025 [21]	3–24 months	3–24 months	All patients	CCS	ACS	ACS	Individualized by DAPT score in PARTHENOPE trial
Viswanathan 2018 [23]	12 months	12 months	All patients	All patients	Not used	Not used	Standard DAPT for 12 months
Gomez 2021 [18]	Not reported	Not reported	Not reported	Not reported	Not reported	Not reported	Not reported
Tao 2021 [22]	Not reported	Not reported	Not reported	Not reported	Not reported	Not reported	Not reported

BP-DES: biodegradable polymer drug-eluting stent; DAPT: dual antiplatelet therapy; PF-DES: polymer-free drug-eluting stent; PCI: percutaneous coronary intervention.

**Table 5:** Summary of findings and certainty assessment for clinical outcomes.

Certainty Assessment							№ of Patients		Effect		Certainty	Importance
№ of Studies	Study Design	Risk of Bias	Inconsistency	Indirectness	Imprecision	Other Considerations	PF-DES	BP-DES	Relative (95% CI)	Absolute (95% CI)		
Myocardial Infarction (follow-up: mean 12 months; assessed with: n)												
6	randomised trials	not serious	not serious ^a^	not serious ^b^	not serious ^c^	none	111/4454 (2.5%)	114/4490 (2.5%)	RR 0.98 (0.77 to 1.25)	1 fewer per 1.000 (from 6 fewer to 6 more)	⨁⨁⨁⨁ High ^a,b,c^	CRITICAL
All-cause death (follow-up: mean 12 months; assessed with: n)												
6	randomised trials	not serious	Serious ^d^	not serious ^e^	Serious ^f^	none	98/4454 (2.2%)	108/4490 (2.4%)	RR 0.89 (0.59 to 1.33)	3 fewer per 1.000 (from 10 fewer to 8 more)	⨁⨁◯◯ Low ^d,e,f^	CRITICAL
Target Lesion Revascularization (follow-up: mean 12 months; assessed with: n)												
6	randomised trials	not serious	not serious	not serious ^g^	Serious ^h^	none	116/3163 (3.7%)	68/3193 (2.1%)	RR 1.51 (0.83 to 2.75)	11 more per 1.000 (from 4 fewer to 37 more)	⨁⨁⨁◯ Moderate ^g,h^	CRITICAL
Stent thrombosis (follow-up: mean 12 months; assessed with: n)												
6	randomised trials	not serious	not serious ^a^	not serious ^g^	Serious ^i^	none	31/4454 (0.7%)	19/4490 (0.4%)	RR 1.69 (0.44 to 6.47)	3 more per 1.000 (from 2 fewer to 23 more)	⨁⨁⨁◯ Moderate ^a,g,i^	CRITICAL
Cardiac death (follow-up: mean 12 months; assessed with: n)												
5	randomised trials	serious ^d^	not serious ^g^	not serious ^f^	serious ^f^	none	58/4384 (1.3%)	66/4406 (1.5%)	RR 0.78 (0.29 to 2.07)	3 fewer per 1.000 (from 11 fewer to 16 more)	⨁⨁◯◯ Low ^d,f,g^	CRITICAL
Target Vessel revascularization (follow-up: mean 12 months; assessed with: n)												
4	randomised trials	not serious	serious ^j^	not serious ^g^	not serious ^k^	none	140/4168 (3.4%)	119/4190 (2.8%)	RR 1.18 (0.90 to 1.56)	5 more per 1.000 (from 3 fewer to 16 more)	⨁⨁⨁◯ Moderate ^g,j,k^	CRITICAL

CI: confidence interval; RR: risk ratio. ^a^ No heterogeneity observed and consistent effect estimates across studies; ^b^ Outcome is clinically direct and consistently defined across relevant populations and interventions; ^c^ Adequate sample size with narrow confidence intervals around a neutral effect estimate; ^d^ Moderate heterogeneity and some variability in populations and event rates; ^e^ All-cause death is a clinically direct and universally relevant endpoint; ^f^ Confidence intervals include both potential benefit and harm, and event rates were relatively low; ^g^ Clinically relevant and directly measured endpoint across comparable populations; ^h^ Adequate number of studies and patients with confidence intervals that do not suggest important benefit or harm; ^i^. Event rates were low and confidence intervals include both possible benefit and harm; ^j^ Moderate to high heterogeneity (I^2^ = 69%) and some variability in revascularization criteria and clinical settings; ^k^ Adequate number of patients and events with reasonably narrow confidence intervals.

**Table 6:** Summary of findings and certainty of evidence for angiographic and OCT outcomes.

Certainty Assessment							№ of Patients		Effect		Certainty	Importance
№ of Studies	Study Design	Risk of Bias	Inconsistency	Indirectness	Imprecision	Other Considerations	PF-DES	BP-DES	Relative (95% CI)	Absolute (95% CI)		
Late Lumen Loss (follow-up: range 6 months to 9 months; assessed with: QCA)												
3	randomised trials	not serious	very serious ^a^	Serious ^b^	Serious ^c^	none	458	469	-	MD 0.11 mm higher (0.32 lower to 0.55 higher)	⨁◯◯◯ Very low ^a,b,c^	IMPORTANT
Binary Restenosis (follow-up: range 6 months to 9 months; assessed with: QCA)												
3	randomised trials	not serious	very serious ^d^	Serious ^e^	Serious ^c^	none	458	469	-	MD 2.66 % higher (8.41 lower to 13.72 higher)	⨁◯◯◯ Very low ^c,d,e^	IMPORTANT
Minimal Lumen Diameter (follow-up: range 6 months to 9 months; assessed with: QCA)												
3	randomised trials	not serious	very serious ^f^	Serious ^g^	Serious ^h^	none	458	469	-	MD 0.11 mm lower (0.38 lower to 0.17 higher)	⨁◯◯◯ Very low ^f,g,h^	IMPORTANT
Malapposed Struts (follow-up: range 1 months to 6 months; assessed with: OCT)												
3	randomised trials	not serious	very serious ^i^	Serious ^j^	Serious ^k^	none	302/25028 (1.2%)	552/28371 (1.9%)	RR 0.81 (0.04 to 14.86)	4 fewer per 1.000 (from 19 fewer to 270 more)	⨁◯◯◯ Very low ^i,j,k^	NOT IMPORTANT
Uncovered Struts (follow-up: range 1 months to 6 months; assessed with: OCT)												
3	randomised trials	not serious	very serious ^l^	Serious ^m^	Serious ^n^	none	2247/12448 (18.1%)	3147/14195 (22.2%)	RR 1.05 (0.59 to 1.88)	11 more per 1.000 (from 91 fewer to 195 more)	⨁◯◯◯ Very low ^l,m,n^	NOT IMPORTANT

CI: confidence interval; MD: mean difference; RR: risk ratio; ^a^ I^2^ = 88%, clinical and methodological heterogeneity across the three studies; no formal exploration feasible; ^b^ Late lumen loss is a surrogate angiographic outcome with limited direct correlation to patient-centered clinical events; ^c^ Wide confidence intervals and small number of studies with modest sample size; ^d^ I^2^ = 89%, different imaging methods and follow-up durations across studies; no formal exploration feasible due to limited number of trials; ^e^ Surrogate outcome assessed with variable imaging modalities (OCT vs. QCA) and follow-up durations (3–9 months); ^f^ I^2^ = 85%, heterogeneous methodologies (QCA vs. OCT), follow-up times, and patient populations; only three studies included; ^g^ MLD is a surrogate angiographic endpoint; ^h^ Only three studies with small sample sizes and wide confidence intervals across heterogeneous designs; ^i^ I^2^ = 88%, only three heterogeneous studies with differing patient populations, OCT timing (1–6 months), and stent platforms; ^j^ Malapposed struts is a surrogate OCT endpoint with variable definitions and measurement timing (1-6 months); ^k^ Small number of studies and patients, with wide confidence intervals and no established threshold of clinical relevance; ^l^ I^2^ = 89%, high variability in clinical setting, OCT timing (16 months), and definitions of strut coverage across only three trials; ^m^ Uncovered struts is a surrogate OCT outcome with variable definitions and follow-up timing across studies (1–6 months); ^n^ Small number of patients, wide confidence intervals, and lack of established threshold for clinical relevance.

## Supplementary Materials

The following supporting information can be downloaded at: https://www.mdpi.com/article/10.3390/biomedicines13061470/s1, Table S1: Medical subject headings (MeSH) and non-MeSH keywords used to search for potential relevant publications; Table S2: Risk of bias summary for randomized studies (RoB2); Table S3: PRISMA checklist for systematic review and meta-analysis; Figure S1: Forest plots showing a sensitivity analysis (excluding Hansen et al., 2022 [13]) for 12-month target lesion revascularization; Figure S2: Forest plots showing a sensitivity analysis (excluding ISAR-TEST 3) for 12-month target lesion revascularization; Figure S3: Forest plots showing sensitivity analysis for 12-month cardiac death (excluding Hansen et al., 2022 [13]); Figure S4: Forest plots showing subgroup analysis of 12-month target lesion revascularization, comparing BioFreedom group (upper panel) vs. non-BioFreedom group (down panel); Figure S5: Forest plots showing subgroup analysis of 12-month cardiac death, comparing BioFreedom group (upper panel) vs non-BioFreedom group (down panel); Figure S6: Funnel plot of 12-months myocardial infarction in patients treated with PF-DES vs. BP-DES; Figure S7: Funnel plot of 12-months all-cause death in patients treated with PF-DES vs. BP-DES; Figure S8: Funnel plot of 12-months target lesion failure in patients treated with PF-DES vs. BP-DES; Figure S9: Funnel plot of 12-months stent thrombosis in patients treated with PF-DES vs. BP-DES; Figure S10: Funnel plot of 12-months cardiac death in patients treated with PF-DES vs. BP-DES; Figure S11: Funnel plot of 12-months target vessel revascularization in patients treated with PF-DES vs. BP-DES; Figure S12: Funnel plot of 24-months myocardial infarction in patients treated with PF-DES vs. BP-DES; Figure S13: Funnel plot of 24-months all-cause death in patients treated with PF-DES vs. BP-DES; Figure S14: Funnel plot of in-stent late lumen loss in patients treated with PF-DES vs. BP-DES; Figure S15: Funnel plot of in-stent binary stenosis in patients treated with PF-DES vs. BP-DES; Figure S16: Funnel plot of in-stent minimal lumen diameter in patients treated with PF-DES vs. BP-DES; Figure S17: Funnel plot of struts malapposition (OCT) in patients treated with PF-DES vs. BP-DES; Figure S18: Funnel plot of number of uncovered struts (OCT) in patients treated with PF-DES vs. BP-DES.

## Abbreviations

The following abbreviations are used in this manuscript:

ACS	acute coronary syndromes
BP-DES	biodegradable polymer drug-eluting stent
CAD	coronary artery disease
CCS	chronic coronary syndromes
DAPT	dual antiplatelet therapy
DES	drug-eluting stent
LLL	late lumen loss
MD	mean difference
MI	myocardial infarction
MLD	mean lumen diameter
OCT	optical coherence tomography
PCI	percutaneous coronary intervention
PF-DES	polymer-free drug-eluting stent
RCT	randomized controlled trial
RR	risk ratio
STEMI	ST-elevated myocardial infarction
TLR	target lesion revascularization
TVR	target vessel revascularization
