# Supplementary material for: Polymer-Free Versus Biodegradable Polymer Drug-Eluting Stents in Coronary Artery Disease: Updated Systematic Review and Meta-Analysis of Clinical, Angiographic, and OCT Outcomes
Source: Biomedicines. 2025 Jun 14;13(6):1470. doi: 10.3390/biomedicines13061470 (PMC12190656; doi:10.3390/biomedicines13061470)
Supplement: Supplementary file 1 [file biomedicines-13-01470-s001.zip › biomedicines-3663413-supplementary.pdf]

biomedicines

MDPI

Supplementary Materials

Table S1. Medical subject headings (MeSH) and non-MeSH keywords used to search for potential relevant publications.

**Concept 1 (drug-eluting stents)**	("Drug-Eluting Stents"[mh]OR "drug-eluting stents"] OR "drug-eluting stent"] OR "Drug coated stent"] OR "Drug coated stents"] OR "DES")
**Concept 2 (intervention)**	("polymer free"[Text Word] OR "without polymer"[Text Word])
**Concept 3 (comparison)**	("biodegradable polymer" OR "bioabsorbable polymer" OR "bioresorbable polymer" OR "bioresorb* polymer" OR "biodegr* polymer" OR "bioabsorb* polymer)

Table S2. Risk of bias summary for randomized studies (RoB2).

Study	Bias from randomization process	Bias due to deviations from intended interventions	Bias due to missing outcome data	Bias in measurement of the outcomes	Bias in selection of the reported result	Overall risk of bias
ISAR-TEST 3	Low risk	Low risk	Low risk	Low risk	Low risk	Low risk
SORT-OUT IX	Low risk	Some concerns	Low risk	Low risk	Low risk	Some concerns
Hansen et al. 2022	Low risk	Low risk	Low risk	Low risk	Low risk	Low risk
Gomez et al. 2021	Low risk	Low risk	Low risk	Low risk	Low risk	Low risk
Hong et al. 2021	Low risk	Low risk	Low risk	Low risk	Low risk	Low risk
Tao et al. 2021	Low risk	Low risk	Low risk	Low risk	Low risk	Low risk
Viswanathan et al. 2018	Low risk	Low risk	Low risk	Some concerns	Low risk	Some concerns
Piccolo et al. 2025	Low risk	Low risk	Low risk	Low risk	Low risk	Low risk
Otaegui et al. 2022	Low risk	Low risk	Low risk	Low risk	Low risk	Low risk

Study or Subgroup	PF-DES Events	PF-DES Total	BP-DES Events	BP-DES Total	Weight	Risk ratio IV, Random, 95% CI
✖ Hansen 2022	2	38	2	42	0.0%	1.11 [0.16 , 7.47]
✔ ISAR-TEST 3	26	201	12	202	22.3%	2.18 [1.13 , 4.19]
✔ Piccolo 2025	20	1051	17	1056	22.6%	1.18 [0.62 , 2.24]
✔ SORT-OUT IX	55	1572	20	1579	25.7%	2.76 [1.66 , 4.59]
✔ Tao 2021	8	216	13	216	18.0%	0.62 [0.26 , 1.45]
✔ Viswanathan 2018	5	85	4	98	11.5%	1.44 [0.40 , 5.20]
**Total (HKSJ[a])**		**3125**		**3151**	**100.0%**	**1.53 [0.73 , 3.20]**
Total events:	114		66			

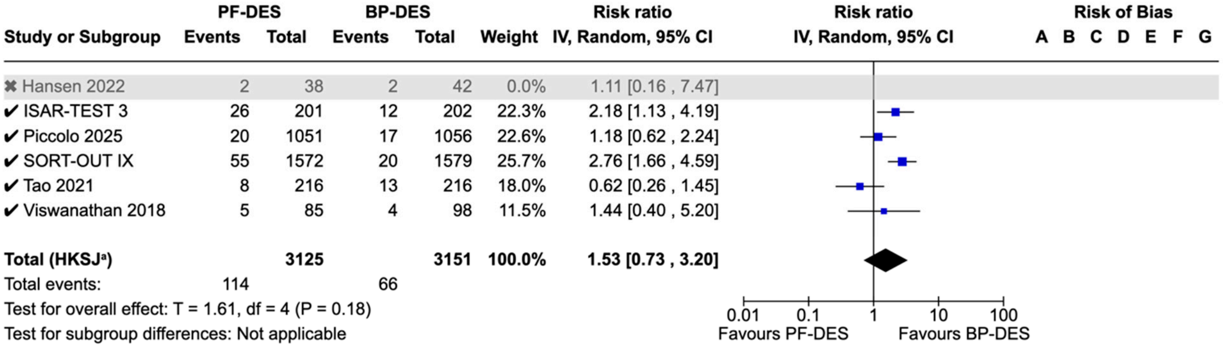

Test for overall effect: T = 1.61, df = 4 (P = 0.18)
Test for subgroup differences: Not applicable
Heterogeneity: Tau² (REML[b]) = 0.23; Chi² = 10.72, df = 4 (P = 0.03); I² = 63%

Footnotes
[a]CI calculated by Hartung-Knapp-Sidik-Jonkman method.
[b]Tau² calculated by Restricted Maximum-Likelihood method.

Figure S1. Forest plots showing a sensitivity analysis (excluding Hansen et al. 2022) for 12-month target lesion revascularization.

Study or Subgroup	PF-DES Events	PF-DES Total	BP-DES Events	BP-DES Total	Weight	Risk ratio IV, Random, 95% CI
**3.1.1 Biofreedom**						
✔ Hansen 2022	2	38	2	42	8.1%	1.11 [0.16 , 7.47]
✖ ISAR-TEST 3	26	201	12	202	0.0%	2.18 [1.13 , 4.19]
✔ Piccolo 2025	20	1051	17	1056	26.5%	1.18 [0.62 , 2.24]
✔ SORT-OUT IX	55	1572	20	1579	29.7%	2.76 [1.66 , 4.59]
✔ Tao 2021	8	216	13	216	21.5%	0.62 [0.26 , 1.45]
✔ Viswanathan 2018	5	85	4	98	14.2%	1.44 [0.40 , 5.20]
**Subtotal (HKSJ[a])**		**2962**		**2991**	**100.0%**	**1.35 [0.64 , 2.87]**
Total events:	90		56			
Test for overall effect: T = 1.11, df = 4 (P = 0.33)						
Heterogeneity: Tau² (REML[b]) = 0.26; Chi² = 10.22, df = 4 (P = 0.04); I² = 60%						
**Total (HKSJ[a])**		**2962**		**2991**	**100.0%**	**1.35 [0.64 , 2.87]**
Total events:	90		56			

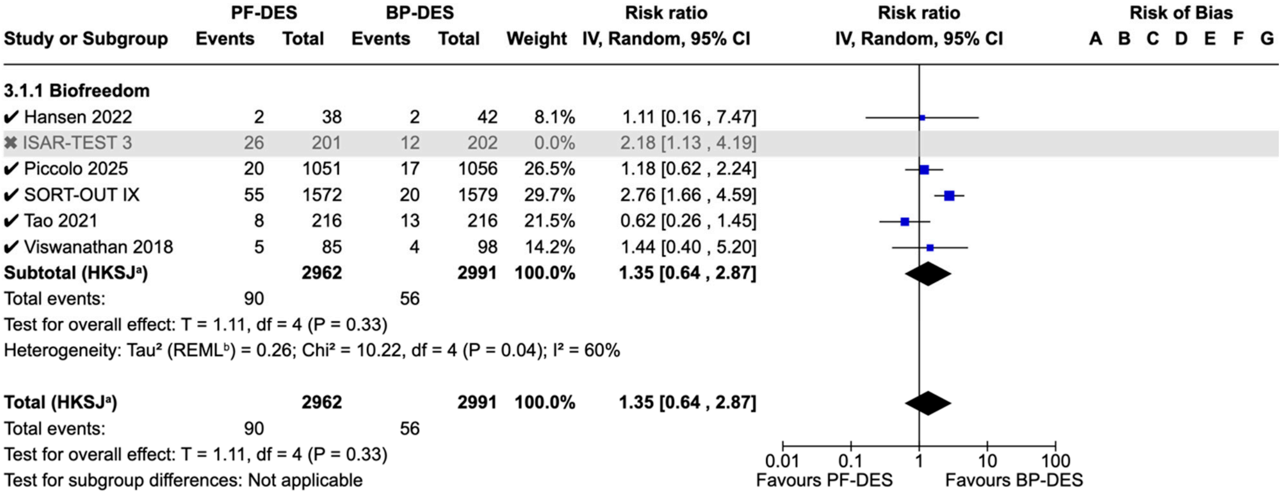

Test for overall effect: T = 1.11, df = 4 (P = 0.33)
Test for subgroup differences: Not applicable
Heterogeneity: Tau² (REML[b]) = 0.26; Chi² = 10.22, df = 4 (P = 0.04); I² = 60%

Footnotes
[a]CI calculated by Hartung-Knapp-Sidik-Jonkman method.
[b]Tau² calculated by Restricted Maximum-Likelihood method.

Figure S2. Forest plots showing a sensitivity analysis (excluding ISAR-TEST 3) for 12-month target lesion revascularization.

Study or Subgroup	PF-DES Events	PF-DES Total	BP-DES Events	BP-DES Total	Weight	Risk ratio IV, Random, 95% CI
✖ Hansen 2022	0	38	0	42		Not estimable
✔ Hong 2021	6	1507	10	1513	22.8%	0.60 [0.22 , 1.65]
✔ Piccolo 2025	36	1051	25	1056	38.2%	1.45 [0.87 , 2.39]
✔ SORT-OUT IX	16	1572	29	1579	34.7%	0.55 [0.30 , 1.02]
✔ Tao 2021	0	216	2	216	4.3%	0.20 [0.01 , 4.14]
**Total (HKSJ[a])**		**4346**		**4364**	**100.0%**	**0.78 [0.29 , 2.07]**
Total events:	58		66			

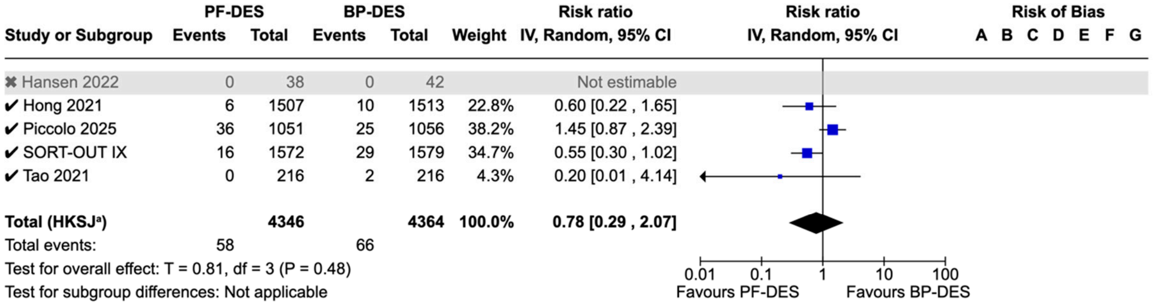

Test for overall effect: T = 0.81, df = 3 (P = 0.48)
Test for subgroup differences: Not applicable
Heterogeneity: Tau² (REML[b]) = 0.23; Chi² = 7.43, df = 3 (P = 0.06); I² = 58%

Footnotes
[a]CI calculated by Hartung-Knapp-Sidik-Jonkman method.
[b]Tau² calculated by Restricted Maximum-Likelihood method.

Figure S3. Forest plots showing sensitivity analysis for 12-month cardiac death (excluding Hansen et al. 2022).

Study or Subgroup	PF-DES Events	PF-DES Total	BP-DES Events	BP-DES Total	Weight	Risk ratio IV, Random, 95% CI
ISAR-TEST 3	26	201	12	202	31.0%	2.18 [1.13 , 4.19]
Piccolo 2025	20	1051	17	1056	31.6%	1.18 [0.62 , 2.24]
Tao 2021	8	216	13	216	23.6%	0.62 [0.26 , 1.45]
Viswanathan 2018	5	85	4	98	13.9%	1.44 [0.40 , 5.20]
**Total (HKSJ[a])**		**1553**		**1572**	**100.0%**	**1.26 [0.53 , 2.97]**
Total events:	59		46			

Test for overall effect: T = 0.86, df = 3 (P = 0.46)
Test for subgroup differences: Not applicable
Heterogeneity: Tau^2 (REML[b]) = 0.14; Chi^2 = 5.39, df = 3 (P = 0.15); I^2 = 47%

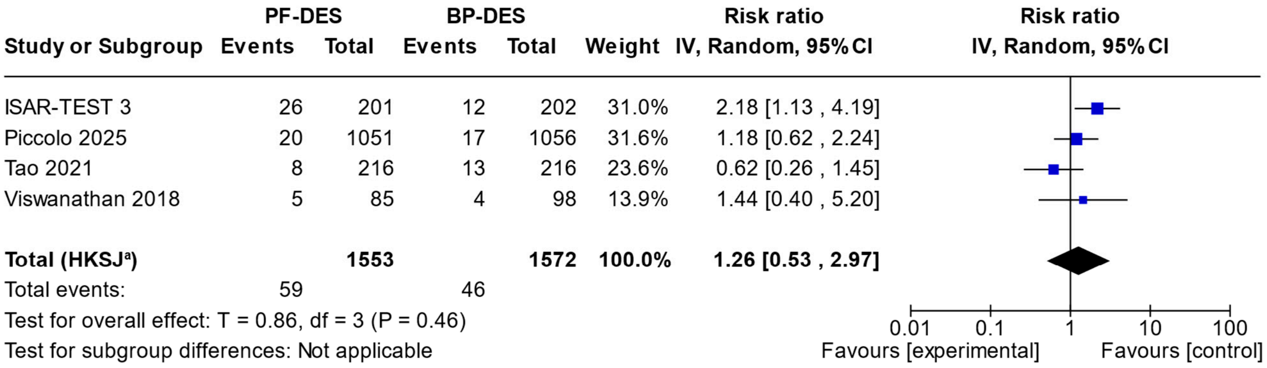

Footnotes
[a]CI calculated by Hartung-Knapp-Sidik-Jonkman method.
[b]Tau^2 calculated by Restricted Maximum-Likelihood method.

Study or Subgroup	PF-DES Events	PF-DES Total	BP-DES Events	BP-DES Total	Weight	Risk ratio IV, Random, 95% CI
Hansen 2022	2	38	2	42	6.6%	1.11 [0.16 , 7.47]
SORT-OUT IX	55	1572	20	1579	93.4%	2.76 [1.66 , 4.59]
**Total (HKSJ[a])**		**1610**		**1621**	**100.0%**	**2.60 [0.15 , 46.59]**
Total events:	57		22			

Test for overall effect: T = 4.21, df = 1 (P = 0.15)
Test for subgroup differences: Not applicable
Heterogeneity: Tau^2 (REML[b]) = 0.00; Chi^2 = 0.83, df = 1 (P = 0.36); I^2 = 0%

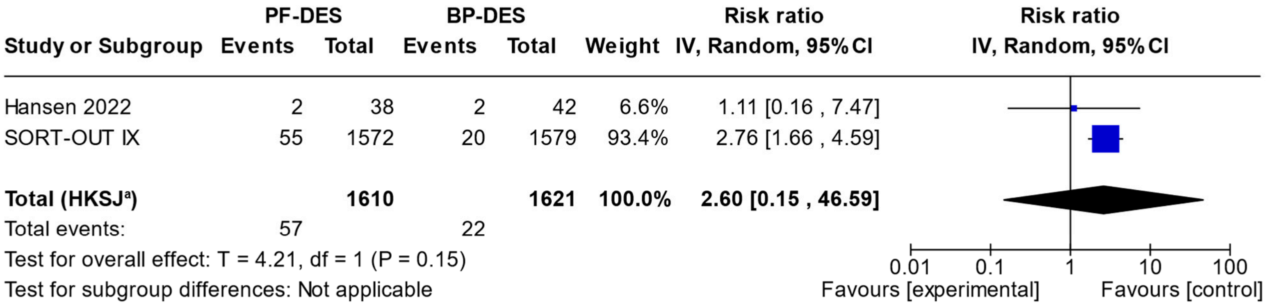

Footnotes
[a]CI calculated by Hartung-Knapp-Sidik-Jonkman method.
[b]Tau^2 calculated by Restricted Maximum-Likelihood method.

Figure S4. Forest plots showing subgroup analysis of 12-month target lesion revascularization, comparing BioFreedom group (upper panel) vs non-BioFreedom group (down panel).

Study or Subgroup	PF-DES Events	PF-DES Total	BP-DES Events	BP-DES Total	Weight	Risk ratio IV, Random, 95% CI
Hansen 2022	0	38	0	42		Not estimable
Hong 2021	6	1507	10	1513	26.5%	0.60 [0.22 , 1.65]
SORT-OUT IX	16	1572	29	1579	73.5%	0.55 [0.30 , 1.02]
**Total (HKSJ[a])**		**3117**		**3134**	**100.0%**	**0.57 [0.35 , 0.90]**
Total events:	22		39			

Test for overall effect: T = 15.43, df = 1 (P = 0.04)
Test for subgroup differences: Not applicable
Heterogeneity: Tau^2 (REML[b]) = 0.00; Chi^2 = 0.02, df = 1 (P = 0.89); I^2 = 0%

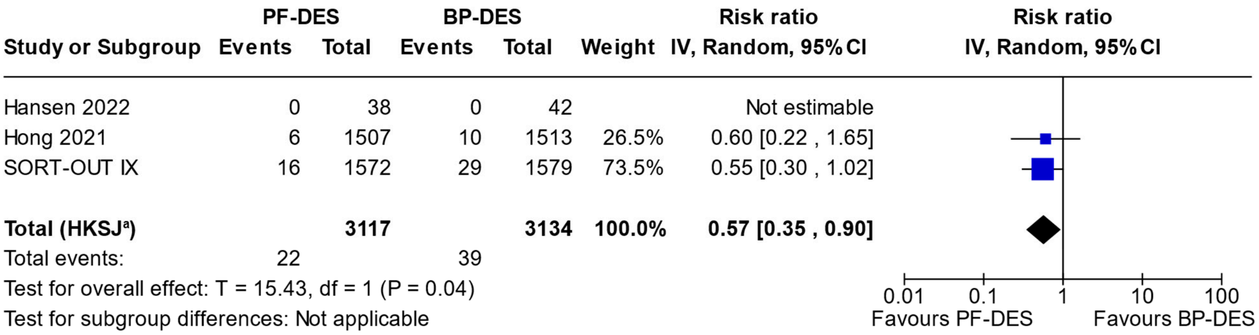

Footnotes
[a]CI calculated by Hartung-Knapp-Sidik-Jonkman method.
[b]Tau^2 calculated by Restricted Maximum-Likelihood method.

Study or Subgroup	PF-DES Events	PF-DES Total	BP-DES Events	BP-DES Total	Weight	Risk ratio IV, Random, 95% CI
Piccolo 2025	36	1051	25	1056	79.7%	1.45 [0.87 , 2.39]
Tao 2021	0	216	2	216	20.3%	0.20 [0.01 , 4.14]
**Total (HKSJ[a])**		**1267**		**1272**	**100.0%**	**0.97 [0.00 , 23942.44]**
Total events:	36		27			

Test for overall effect: T = 0.04, df = 1 (P = 0.97)
Test for subgroup differences: Not applicable
Heterogeneity: Tau² (REML[b]) = 0.73; Chi² = 1.59, df = 1 (P = 0.21); I² = 37%

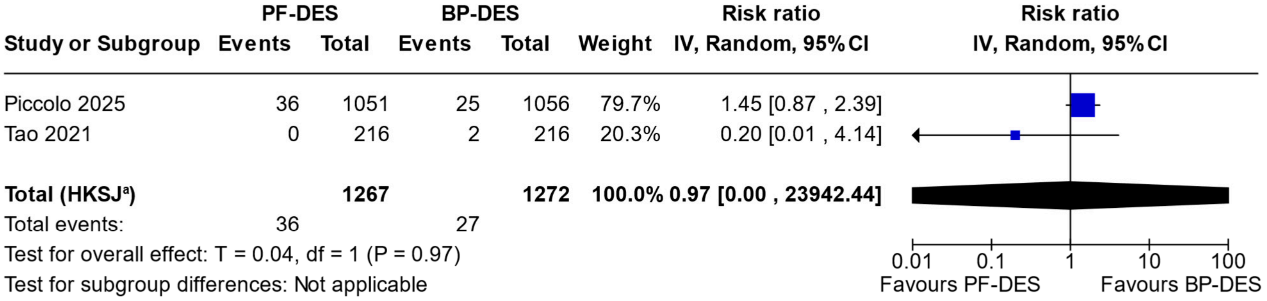

Footnotes
[a]CI calculated by Hartung-Knapp-Sidik-Jonkman method.
[b]Tau² calculated by Restricted Maximum-Likelihood method.

Figure S5. Forest plots showing subgroup analysis of 12-month cardiac death, comparing BioFreedom group (upper panel) vs non-BioFreedom group (down panel).

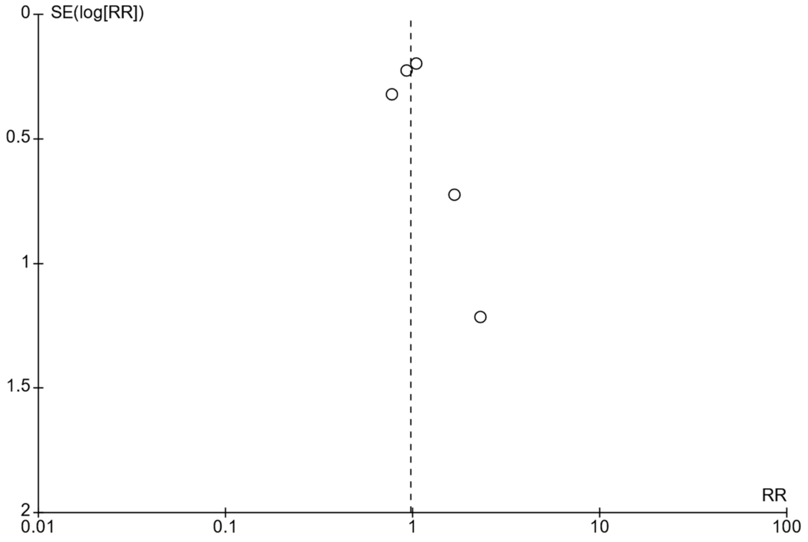

Figure S6. Funnel plot of 12-months myocardial infarction in patients treated with PF-DES vs BP-DES.

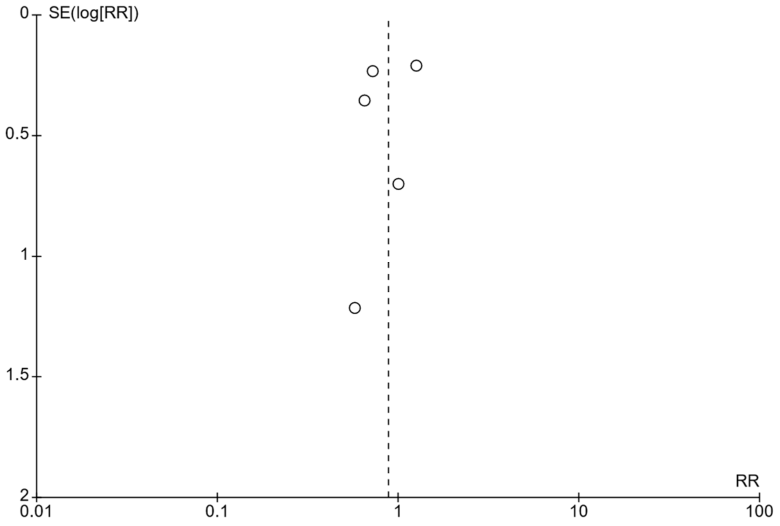

Figure S7. Funnel plot of 12-months all-cause death in patients treated with PF-DES vs BP-DES.

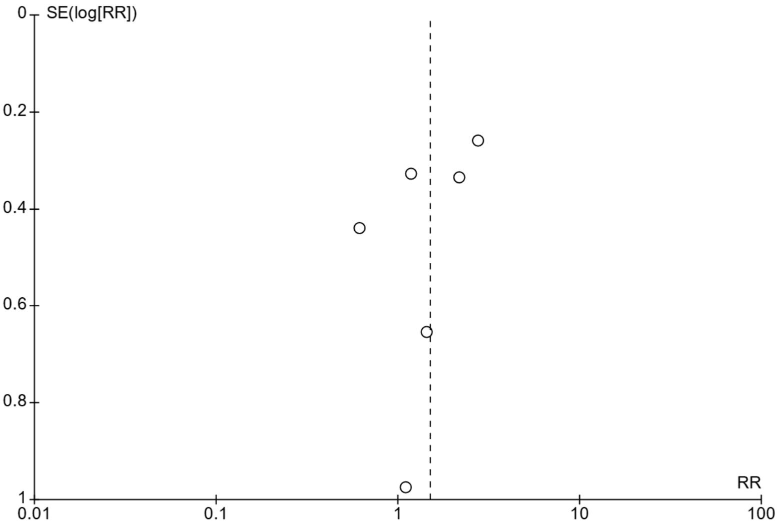

Figure S8. Funnel plot of 12-months target lesion failure in patients treated with PF-DES vs BP-DES.

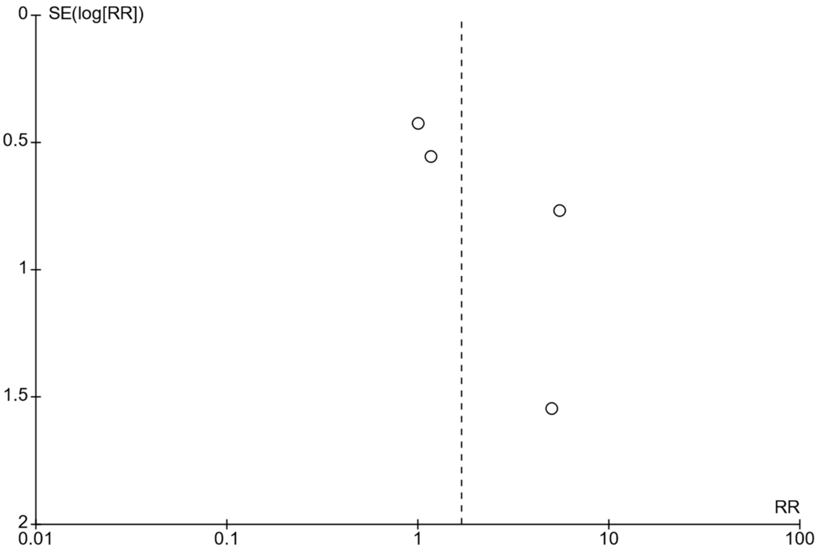

Figure S9. Funnel plot of 12-months stent thrombosis in patients treated with PF-DES vs BP-DES.

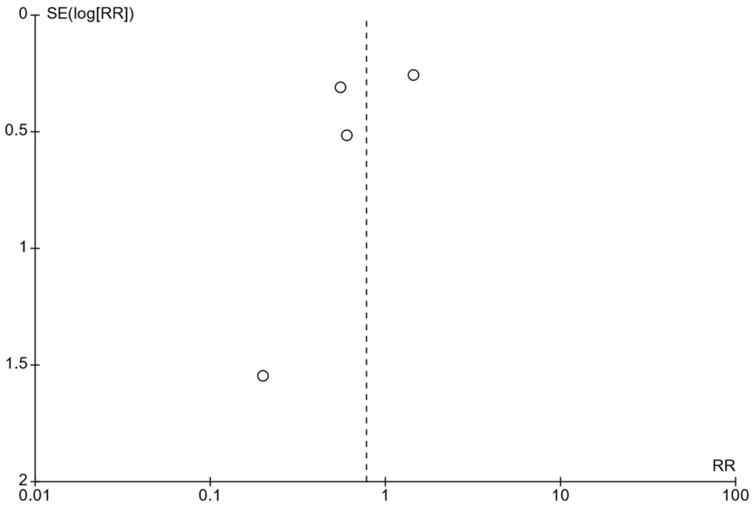

Figure S10. Funnel plot of 12-months cardiac death in patients treated with PF-DES vs BP-DES.

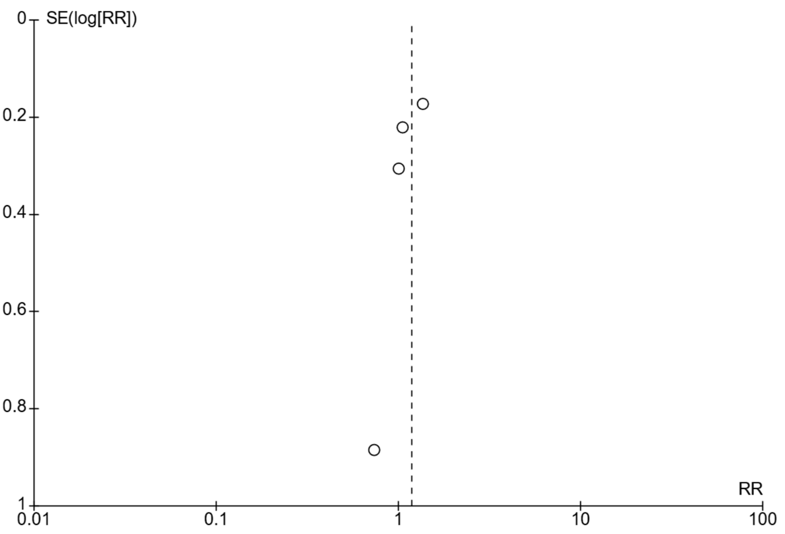

Figure S11. Funnel plot of 12-months target vessel revascularization in patients treated with PF-DES vs BP-DES.

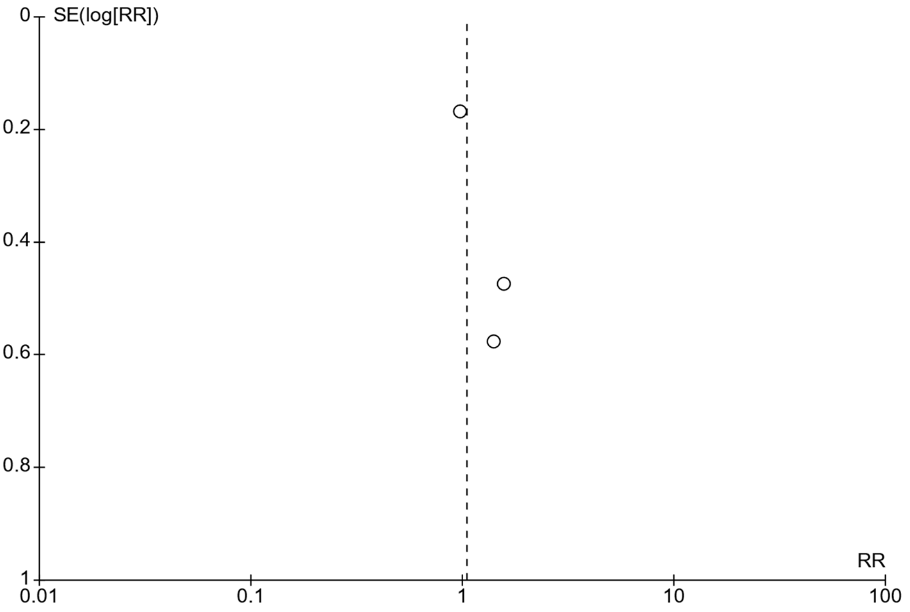

Figure S12. Funnel plot of 24-months myocardial infarction in patients treated with PF-DES vs BP-DES.

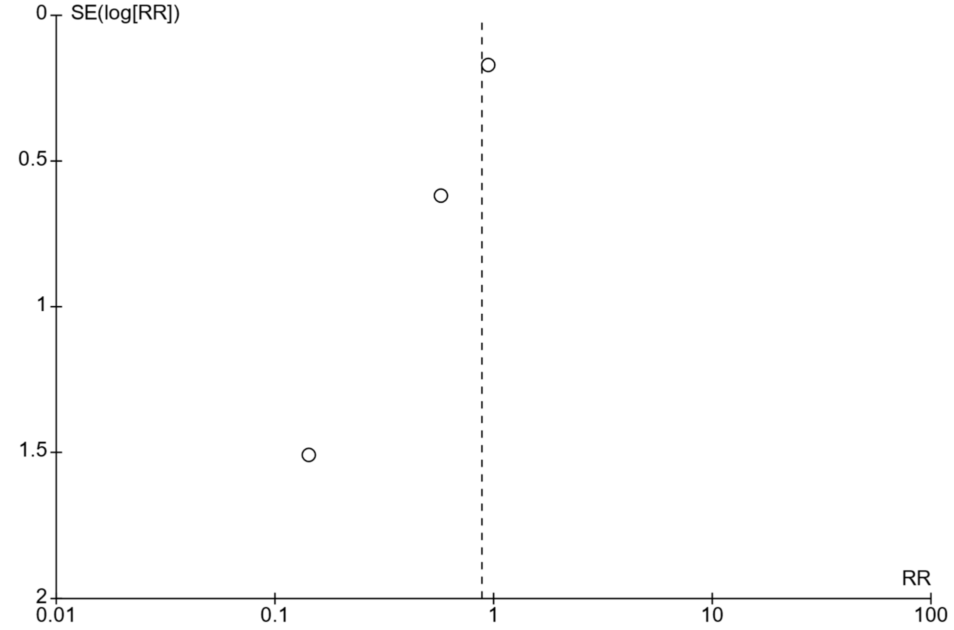

Figure S13. Funnel plot of 24-months all-cause death in patients treated with PF-DES vs BP-DES.

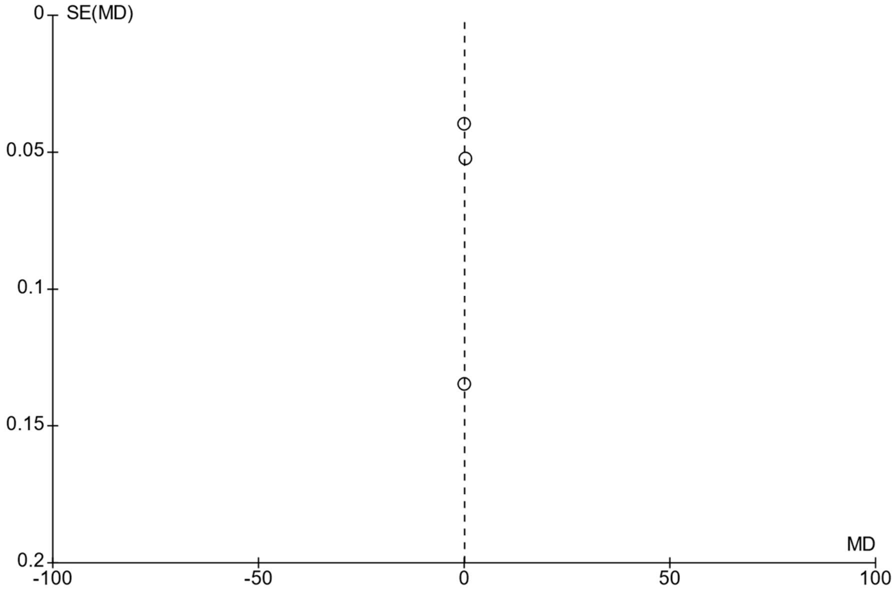

Figure S14. Funnel plot of in- stent late lumen loss in patients treated with PF-DES vs BP-DES.

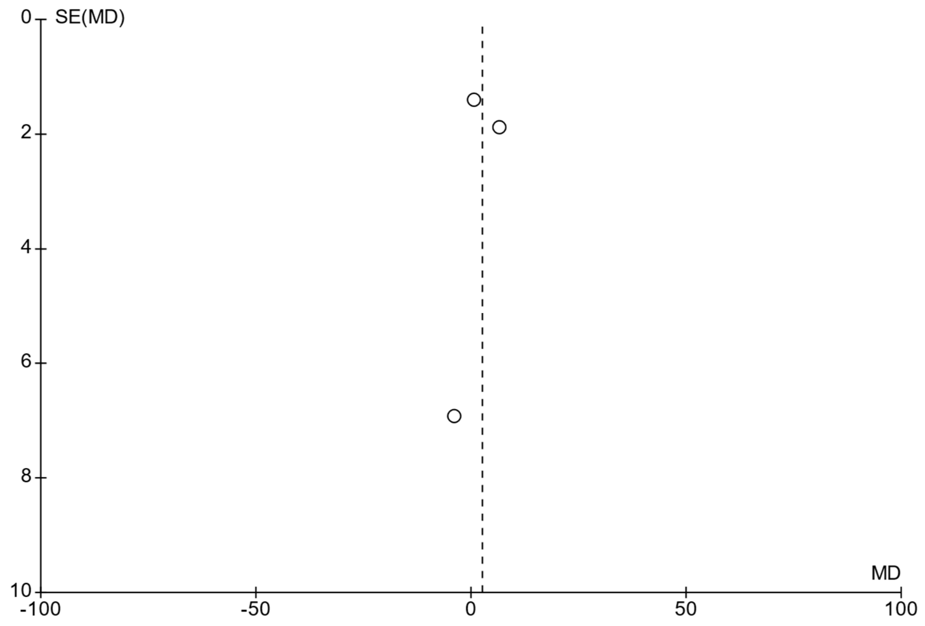

Figure S15. Funnel plot of in-stent binary stenosis in patients treated with PF-DES vs BP-DES.

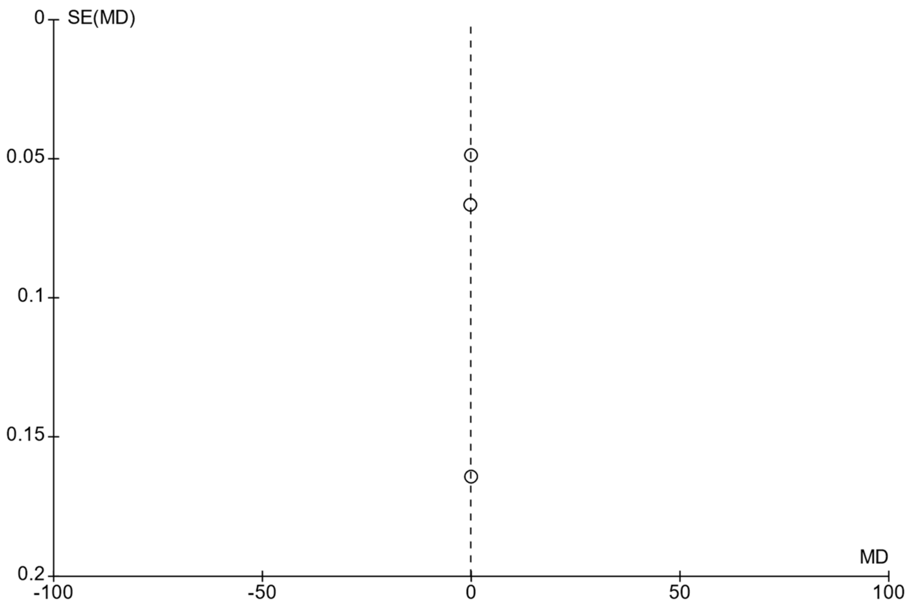

Figure S16. Funnel plot of in-stent minimal lumen diameter in patients treated with PF-DES vs BP-DES.

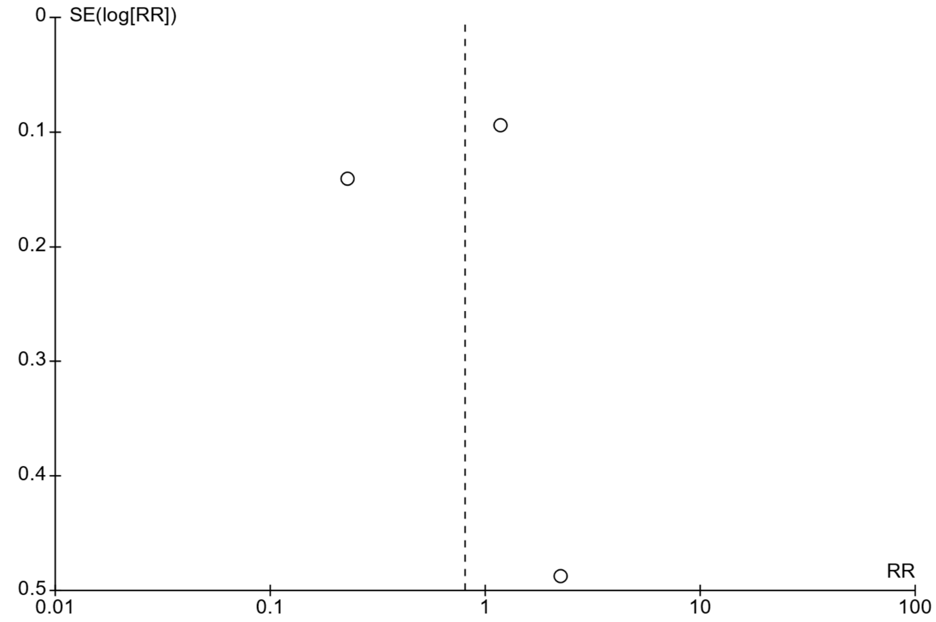

Figure S17. Funnel plot of struts malapposition (OCT) in patients treated with PF-DES vs BP-DES.

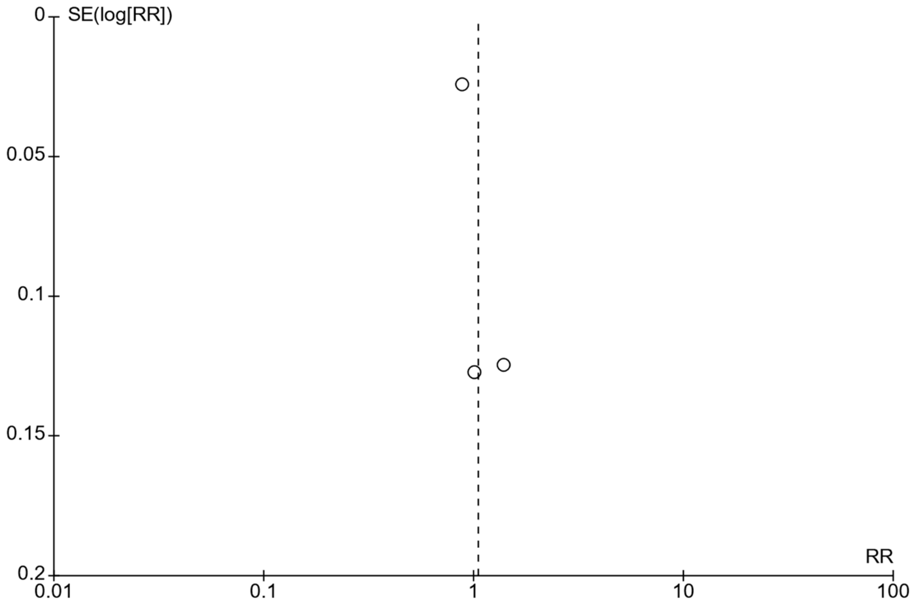

Figure S18. Funnel plot of number of uncovered struts (OCT) in patients treated with PF-DES vs BP-DES.

Table S3. PRISMA checklist for systematic review and meta-analysis.

Section and Topic	Item #	Checklist item	Location where item is reported
**TITLE**			
Title	1	Identify the report as a systematic review.	Title page, line 3
**ABSTRACT**			
Abstract	2	See the PRISMA 2020 for Abstracts checklist.	Page 1, abstract section
**INTRODUCTION**			
Rationale	3	Describe the rationale for the review in the context of existing knowledge.	Page 2, Introduction, lines 49–80
Objectives	4	Provide an explicit statement of the objective(s) or question(s) the review addresses.	Page 2, line 75–80: Explicit objective stated
**METHODS**			
Eligibility criteria	5	Specify the inclusion and exclusion criteria for the review and how studies were grouped for the syntheses.	Page 2–3, Eligibility criteria, lines 88–97
Information sources	6	Specify all databases, registers, websites, organisations, reference lists and other sources searched or consulted to identify studies. Specify the date when each source was last searched or consulted.	Page 3, Information sources, lines 102–106
Search strategy	7	Present the full search strategies for all databases, registers and websites, including any filters and limits used.	Page 3, Search strategy in main text and full search in

Section and Topic	Item #	Checklist item	Location where item is reported
			Supplementary Table S1
Selection process	8	Specify the methods used to decide whether a study met the inclusion criteria of the review, including how many reviewers screened each record and each report retrieved, whether they worked independently, and if applicable, details of automation tools used in the process.	Page 3, Study selection, lines 109–114
Data collection process	9	Specify the methods used to collect data from reports, including how many reviewers collected data from each report, whether they worked independently, any processes for obtaining or confirming data from study investigators, and if applicable, details of automation tools used in the process.	Page 3, Data collection pro-cess, lines 115–119
Data items	10a	List and define all outcomes for which data were sought. Specify whether all results that were compatible with each outcome domain in each study were sought (e.g. for all measures, time points, analyses), and if not, the methods used to decide which results to collect.	Page 3, Data items, lines 120–123
	10b	List and define all other variables for which data were sought (e.g. participant and intervention characteristics, funding sources). Describe any assumptions made about any missing or unclear information.	Page 3, Data items, lines 120–124
Study risk of bias assessment	11	Specify the methods used to assess risk of bias in the included studies, including details of the tool(s) used, how many reviewers assessed each study and whether they worked independently, and if applicable, details of automation tools used in the process.	Page 3, Risk of bias assess-ment, lines 125–128
Effect measures	12	Specify for each outcome the effect measure(s) (e.g. risk ratio, mean difference) used in the synthesis or presentation of results.	Page 3, Data synthesis and statistical analy-sis, lines 129–133

Section and Topic	Item #	Checklist item	Location where item is reported
Synthesis methods	13a	Describe the processes used to decide which studies were eligible for each synthesis (e.g. tabulating the study intervention characteristics and comparing against the planned groups for each synthesis (item #5)).	Page 3, Eligibility criteria and synthesis methods, lines 88–91 and 129–130
	13b	Describe any methods required to prepare the data for presentation or synthesis, such as handling of missing summary statistics, or data conversions.	Page 3, line 135: No imputation or conversions
	13c	Describe any methods used to tabulate or visually display results of individual studies and syntheses.	Figures 2–6 in main text, and Supplementary Tables
	13d	Describe any methods used to synthesize results and provide a rationale for the choice(s). If meta-analysis was performed, describe the model(s), method(s) to identify the presence and extent of statistical heterogeneity, and software package(s) used.	Page 3, lines 129–133: Random-effects model, RevMan used
	13e	Describe any methods used to explore possible causes of heterogeneity among study results (e.g. subgroup analysis, meta-regression).	Page 4, Additional Analyses section, lines 140–159
	13f	Describe any sensitivity analyses conducted to assess robustness of the synthesized results.	Page 4 and 6, sensitivity analyses detailed in lines 229–247

Section and Topic	Item #	Checklist item	Location where item is reported
			and Supplementary Figures S3–S7
Reporting bias assessment	14	Describe any methods used to assess risk of bias due to missing results in a synthesis (arising from reporting biases).	Page 4, Reporting Bias, lines 137–139 and Page 6, lines 248–257
Certainty assessment	15	Describe any methods used to assess certainty (or confidence) in the body of evidence for an outcome.	Not assessed
**RESULTS**			
Study selection	16a	Describe the results of the search and selection process, from the number of records identified in the search to the number of studies included in the review, ideally using a flow diagram.	Page 6–7, Study selection and PRISMA flow diagram (Figure 1)
	16b	Cite studies that might appear to meet the inclusion criteria, but which were excluded, and explain why they were excluded.	Page 6–7, lines 164–167 and Figure 1: Reasons for exclusion listed
Study characteristics	17	Cite each included study and present its characteristics.	Page 6 and Tables 1–3
Risk of bias in studies	18	Present assessments of risk of bias for each included study.	Page 6, lines 248–252 and Supplementary

Section and Topic	Item #	Checklist item	Location where item is reported
			Table S2
Results of individual studies	19	For all outcomes, present, for each study: (a) summary statistics for each group (where appropriate) and (b) an effect estimate and its precision (e.g. confidence/credible interval), ideally using structured tables or plots.	Figures 2–6, individual study results presented visually
Results of syntheses	20a	For each synthesis, briefly summarise the characteristics and risk of bias among contributing studies.	Page 6, lines 248–252: Study characteristics and RoB summarized
	20b	Present results of all statistical syntheses conducted. If meta-analysis was done, present for each the summary estimate and its precision (e.g. confidence/credible interval) and measures of statistical heterogeneity. If comparing groups, describe the direction of the effect.	Figures 2–6: Summary effect sizes, CIs, heterogeneity
	20c	Present results of all investigations of possible causes of heterogeneity among study results.	Page 6, lines 229–247: Subgroup analyses
	20d	Present results of all sensitivity analyses conducted to assess the robustness of the synthesized results.	Page 6, lines 229–247: Sensitivity analyses
Reporting biases	21	Present assessments of risk of bias due to missing results (arising from reporting biases) for each synthesis assessed.	Page 6, lines 248–257: Funnel plots, visual inspection

Section and Topic	Item #	Checklist item	Location where item is reported
Certainty of evi-dence	22	Present assessments of certainty (or confidence) in the body of evidence for each outcome assessed.	Not assessed
**DISCUSSION**			
Discussion	23a	Provide a general interpretation of the results in the context of other evidence.	Pages 15–16, Discussion, lines 399–477
	23b	Discuss any limitations of the evidence included in the review.	Page 16, lines 449–456
	23c	Discuss any limitations of the review processes used.	Page 16, lines 449–456
	23d	Discuss implications of the results for practice, policy, and future research.	Page 16–17, lines 471–477
**OTHER INFORMATION**			
Registration and protocol	24a	Provide registration information for the review, including register name and registration number, or state that the review was not registered.	Page 2, line 86
	24b	Indicate where the review protocol can be accessed, or state that a protocol was not prepared.	Protocol not publicly accessi-ble
	24c	Describe and explain any amendments to information provided at registration or in the protocol.	No protocol amendments
Support	25	Describe sources of financial or non-financial support for the review, and the role of the funders or sponsors in the review.	Page 16, line 485: ‘No external funding
Competing	26	Declare any competing interests of review authors.	Page 16, line

Section and Topic	Item #	Checklist item	Location where item is reported
interests			486: ‘No conflicts of interest
Availability of data, code and other materials	27	Report which of the following are publicly available and where they can be found: template data collection forms; data extracted from included studies; data used for all analyses; analytic code; any other materials used in the review.	Page 16, line 478
